# *MYCN* induces cell-specific tumorigenic growth in *RB1*-proficient human retinal organoid and chicken retina models of retinoblastoma

**DOI:** 10.1038/s41389-022-00409-3

**Published:** 2022-06-21

**Authors:** Maria K. E. Blixt, Minas Hellsand, Dardan Konjusha, Hanzhao Zhang, Sonya Stenfelt, Mikael Åkesson, Nima Rafati, Tatsiana Tararuk, Gustav Stålhammar, Charlotta All-Eriksson, Henrik Ring, Finn Hallböök

**Affiliations:** 1 Department of Immunology, Genetics, and Pathology, Uppsala University, Uppsala, Sweden; 2 National Bioinformatics Infrastructure Sweden, Uppsala University, SciLifeLab, Department of Medical Biochemistry and Microbiology, Uppsala, Sweden; 3 St. Erik Eye Hospital, Department of Clinical Neuroscience, Karolinska Institute, Stockholm, Sweden

**Keywords:** Cancer models, Embryonal neoplasms, Paediatric cancer, CNS cancer

## Abstract

Retinoblastoma is a rare, intraocular paediatric cancer that originates in the neural retina and is most frequently caused by bi-allelic loss of *RB1* gene function. Other oncogenic mutations, such as amplification and increased expression of the *MYCN* gene, have been found even with proficient RB1 function. In this study, we investigated whether MYCN over-expression can drive carcinogenesis independently of *RB1* loss-of-function mutations. The aim was to elucidate the events that result in carcinogenesis and identify the cancer cell-of-origin. We used the chicken retina, a well-established model for studying retinal neurogenesis, and established human embryonic stem cell-derived retinal organoids as model systems. We over-expressed *MYCN* by electroporation of piggyBac genome-integrating expression vectors. We found that over-expression of MYCN induced tumorigenic growth with high frequency in RB1-proficient chicken retinas and human organoids. In both systems, the tumorigenic cells expressed markers for undifferentiated cone photoreceptor/horizontal cell progenitors. The over-expression resulted in metastatic retinoblastoma within 7–9 weeks in chicken. Cells expressing MYCN could be grown in vitro and, when orthotopically injected, formed tumours that infiltrated the sclera and optic nerve and expressed markers for cone progenitors. Investigation of the tumour cell phenotype determined that the potential for neoplastic growth was embryonic stage-dependent and featured a cell-specific resistance to apoptosis in the cone/horizontal cell lineage, but not in ganglion or amacrine cells. We conclude that MYCN over-expression is sufficient to drive tumorigenesis and that a cell-specific resistance to apoptosis in the cone/horizontal cell lineage mediates the cancer phenotype.

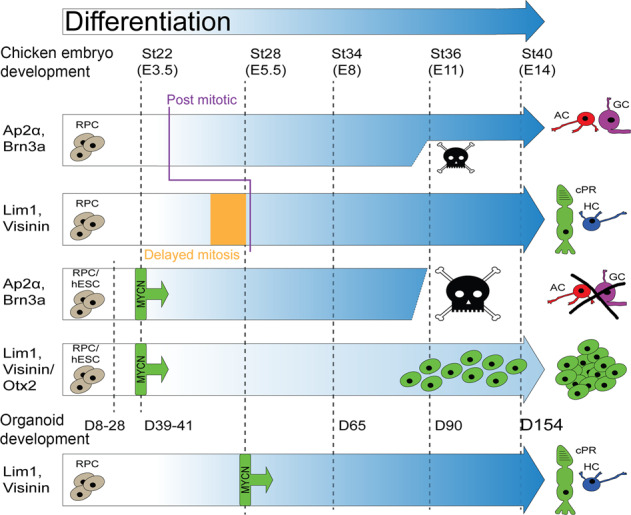

## Introduction

Cell death occurs naturally in the retina and is essential for morphogenesis, adaptive removal of post-mitotic neurons [1–3], and protection against cell cycle aberrations and DNA damage [4]. Mechanisms for the execution of developmental cell death depend on the specific process during which the cell death programme is activated. Proliferating neural stem and undifferentiated progenitor cells respond to DNA damage with cell cycle arrest, DNA repair, or apoptosis. Activation of damage-responses in differentiating progenitors mostly leads to apoptosis whilst mature, post-mitotic neurons are not affected [5]. Oncogenic mutations in cells that are not repaired or removed may lead to neoplastic cell growth but cancer rarely affects neurons because they are post-mitotic. However, when oncogenic mutations strike neuronal progenitors, cancers of neuronal origin may develop. Retinoblastoma is a childhood cancer that originates in the neural retina and expression-profiling of tumours, as well as experimental studies, show that the cellular origin for retinoblastoma often lies in the lineage of cone photoreceptors (cPRs) [6]. Developmentally, cPRs and horizontal cells (HCs) are derived from the same multipotent, fate-restricted progenitor cells [7–10] and in a mouse model of retinoblastoma, the tumour had HC or HC progenitor features [11]. Interestingly, cPRs and HCs are exceptions to the rule that developing retinal neurons undergo extensive developmental cell death [12, 13]. Additionally, these progenitors perform a delayed, amplifying mitosis in a semi-differentiated state as their terminal mitosis before becoming post-mitotic [7, 10, 14]. Furthermore, HC progenitors divide and escape apoptosis during this final cell cycle even with DNA damage [15–18]. It has therefore been suggested that it is the properties of this progenitor cell lineage that renders it more susceptible to neoplastic transformation into retinoblastoma, compared to other retinal neurons. Retinoblastoma carcinogenesis is strongly associated with *RB1* mutations and develops with high frequency after bi-allelic loss-of-function of the *RB1* gene (*RB1*^−/−^) [19–21]. Copy-number amplifying mutations of the *MYCN* gene (MYCN^A^) causing *MYCN* over-expression have been found in retinoblastomas [22–25]. Some of the retinoblastomas with MYCN^A^ have intact *RB1* genes (*RB1*^*+/+, +/−*^) with proficient expression of Rb1 [24, 25]. This suggests that retinoblastoma carcinogenesis can progress in a functionally normal *RB1* genetic background and that other oncogenic mutations, such as MYCN^A^, drives tumorigenic growth. Arguments against this notion are that mice do not develop retinoblastoma with *MYCN* over-expression unless they also have deficient *Rb1* expression [26] and that RB1 functions may be inactivated in the *RB1*-proficient, MYCN^A^ retina by phosphorylation or by other means than gene mutation [27].

In this study, we over-expressed *MYCN* in the developing chicken retina and in human embryonic stem cell (hESC)-derived retinal organoids to model MYCN^A^-driven retinoblastoma in *RB1*^*+/+*^ genetic background. We focused the analyses on the formation of cPR/HC progenitors with the aim of determining how the cancer phenotype is established. We also wanted to determine whether *MYCN* over-expression can generate retinoblastoma with high frequency in *RB1*-proficient retina. We used the transposon-mediated, genome-integrating piggyBac expression system, with GFP as a reporter, to generate stable *MYCN* over-expression in the chicken retina and in human organoids. In both systems, *MYCN* expression produced clusters of atypical, mitotic retinal cells that exhibited anaplastic properties and expressed markers for cPR and HC progenitors, but not for amacrine cells (AC) or retinal ganglion cells (GC). In chicken, cells with AC and GC markers initially expressed the *MYCN* construct, but were removed by apoptosis during stages that coincided with their naturally occurring cell death. The surviving neoplastic cells proliferated continuously and formed metastatic tumours that infiltrated the optic nerve and expanded extra-ocularly within 7–9 weeks. Our results also showed that the retinal organoids produced cells that resembled the chicken cluster cells; they continued to proliferate and had markers for cPR/HC progenitors, but not for ACs or GCs. We conclude that *MYCN*-expression generates anaplastic neoplasia in *RB1*-proficient retinal cells, arising from cell-specific resistance to naturally occurring death combined with the transforming activity of *MYCN*.

## Results

### Expression of *MYCN* in the embryonic chicken retina generated clusters of proliferating cells with markers of cPRs or HC progenitor cells

To mimic *MYCN* over-expression after copy number *MYCN*-mutations in retinoblastoma [24, 25], we used the genome-integrating piggyBac expression system to generate stable and cell-ubiquitous expression of human *MYC*-sequences in chicken retinal cells. The stability of MYC transcription factors is low and phosphorylation of residue T58 increases proteasomal degradation [28]. The T58A mutant variants of MYC disrupt the proteasome signal, thus potentiating their effects, producing a robust cancer phenotype [29]. Both *MYCN* and c-*MYC* with and without the oncogenic T58A mutation were used. The *MYC* transgene-expression was identified by the expression of GFP from the same bi-cistronic transcription unit (Table S1 in Supplementary Material).

**Fig. 1:**
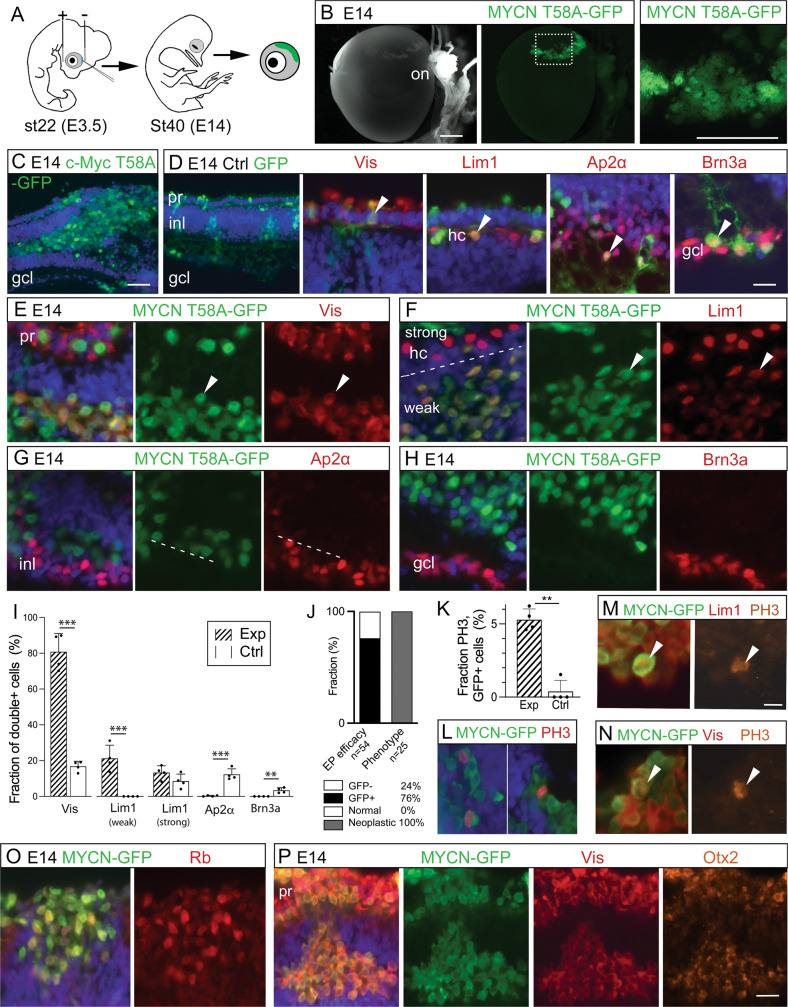
Effects of MYC over-expression in chicken retina. Fluorescence micrographs of retinal cross sections and bar graphs of cell counting of in ovo electroporated st22/E3.5 chicken embryos with immunohistochemical analysis at st40/E14. GFP epifluorescence indicates transgenic cells in control (Ctrl) and experimental groups (Exp, electroporated with human *MYCN* or c-*MYC* with or without the oncogenic T58A mutation). **A** Schematic illustration of subretinal plasmid injection and electroporation. **B** Representative micrograph of GFP fluorescence for the Exp group. Dashed box is magnified in the right panel. Micrographs of **C** GFP positive cells in c-*MYC*^T58A^ electroporated retina (note the cell-cluster phenotype and disrupted retinal lamination), and **D** control-electroporated retina with IR for GFP, Visinin (Vis), GFP, Lim1, GFP, Ap2α, and GFP, Brn3a double-positive cells. Micrographs of experimental *MYCN*^T58A^ retina with IR for **E** GFP, Visinin, **F** GFP, Lim1 (note weak staining over cluster cells and strong staining in HCs delineated by a dashed line), **G** GFP, Ap2α, and **H** GFP, Brn3a. **I** Bar graph with counts of GFP and Vis, Lim1, Ap2α, or Brn3a double-positive cells in st40/E14 retina, Ctrl vs Exp (*MYCN*) groups. **J** Stacked bar graph of the efficacy of electroporation and the penetrance of the cluster-phenotype after *MYCN*-electroporation. **K** Bar graph with counts and **L** micrographs with GFP, phospho-histone 3 (PH3) double-positive cells in Ctrl vs Exp groups. Micrographs of **M** GFP, PH3, Lim1 triple-positive and **N** GFP, PH3, Vis triple-positive cells in Exp groups. Note the PH3 IR over mitotic figures in panels **L**–**N** Micrographs of **O** GFP, Rb double-positive and **P** GFP, Vis, Otx2 triple-positive cells in Exp group st40/E14 retina. Mean ± SD, **p < 0.01, ***p < 0.005, **I** ANOVA *n* = 4, 6 550 GFP+ cells analysed, and **K** Student’s *t*-test *n* = 4, 1 587 GFP+ cells analysed. Arrowheads exemplify double- and triple-positive cells. E embryonic day, gcl ganglion cell layer, hc horizontal cell, inl inner nuclear layer, IR immunoreactivity, on optic nerve, PH3 phospho-histone 3, pr photoreceptor, st Hamburger & Hamilton developmental stage, Vis Visinin. Scale bars in **B**, 500 µm; in **C**, 200 µm also applicable to left panel in (**D**), in **D**, 25 µm also for **E**–**H**, **M**, **N**, **P**; in **O**, 10 µm, and **P**, 50 µm.

The MYC-vectors were introduced in st22/E3.5 embryonic retina by *in ovo* electroporation, targeting multipotent retinal progenitor cells (Fig. 1A). The effects were analysed at st40/E14, a time when the neurons have formed and are positioned in their prospective mature retinal layers [30]. Large clusters of GFP positive (+) cells with round somas without neurites were seen at the site of electroporation in the experimental groups with *MYCN*, c-*MYC*, and their T58A variants. The cell clusters disrupted the retinal morphology (Figs. 1B, C and S1). GFP-controls produced green cells within a normal retina without any clusters. The control GFP+ cells had normal morphologies and expressed markers for PRs; Visinin, HCs; Lim1/2 (Lim1), ACs; Ap2α, and GCs; Brn3a (Fig. 1D). Visinin, a homologue of recoverin [31], is a marker for immature PRs and Visinin immunoreactivity (IR) is seen in cones [32, 33]. The *MYCN*^T58A^ and c-*MYC*^T58A^ expression induced the formation of cell clusters with Visinin and Lim1 IR, but not Ap2α or Brn3a when analysed at st40/E14 (Fig. 1E–H). Lim1 IR is notably stronger in mature HCs than in progenitors [34] and can be identified by their position. Lim1 IR was stronger in naïve GFP negative HCs than in GFP+ cluster cells (weak Lim1 IR, Figs. 1F and S1). A significant accumulation of GFP, Visinin and GFP, Lim1_(weak)_ double-positive (GFP+Lim1+) cells was observed in the clusters compared to control. The number of GFP+Ap2α+ and GFP+Brn3a+ cells was significantly lower in the experimental group than the control group (Fig. 1I). There was a 100% penetrance of the cell-cluster phenotype in successfully electroporated animals (Fig. 1J). These results imply that over-expression of *MYCN* or c-*MYC* at st22/E3.5 generates a phenotype at st40/E14 featuring cell clusters with marker-IR for developing progenitors of PRs and HCs, but not for ACs or GCs.

The experimental group had significantly more GFP+phospho-histone H3+ (PH3+) cells than control (Figs. 1K, L and S1). There were GFP, PH3, and Visinin or Lim1 triple-positive mitotic figures (Fig. 1M, N) as well as GFP+Rb+ cells within the clusters (Fig. 1O). The GFP+Visinin+ cells were also IR for Otx2, a transcription factor that promotes cPR development [35] (Fig. 1P). Progenitors that give rise to both cPRs and HCs [7, 10] have weak Lim1 IR [36], as the cluster cells do. These results show that the cluster-cells proliferate and exhibit features of a cPR/HC progenitor.

Interestingly, the cluster-phenotype observed at st40/E14 was identical when expression of *MYCN*^T58A^ was regulated by a 208 bp RXRγ gene sequence [7], specific for cPR/HC progenitors, as when expressed cell-ubiquitously (Fig. S1). This finding lends support to the hypothesis that the cluster-cells are derived from the cPR/HC progenitor lineage. It also raised the question of how this phenotype was established. To explore this, we studied the timing and formation of the *MYC*-induced clusters.

### Generation of the *MYCN*-induced cluster-cells is stage-specific and involves cell-specific resistance to apoptosis

**Fig. 2:**
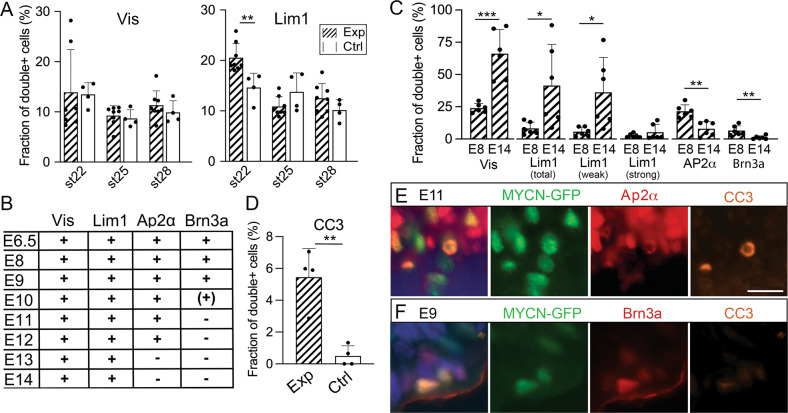
Stage- and cell-specific responses to over-expression of *MYCN*, *MYCN*^T58A^, and *c-MYC*^T58A^ in chicken retina. Fluorescence micrographs and cell counts of retinas electroporated with *MYC* constructs and analysed at various stages. GFP epifluorescence indicate *MYC* over-expressing cells. Effects of over-expression on retinal cell types over time were analysed. **A** Bar graphs with fractions of GFP, Lim1 and GFP, Visinin (Vis) double-positive cells in experimental (Exp) and control (Ctrl) group retinas electroporated at st22/E3.5, st25/E4.5, or st28/E5.5 and analysed after 48 h. Experimental group consisted of animals electroporated with c-*MYC*^T58A^ or *MYCN*^T58A^. See also Fig. S2A. **B** Table displaying the estimation of presence (+) or absence (−) of GFP and Vis, Lim1, Ap2α, or Brn3a double-positive cells in retinas electroporated with c-*MYC*^T58A^ or *MYCN*^T58A^ at st22/E3.5 and analysed at st30/E6.5-st40/E14. **C** Bar graphs with fractions of GFP and Vis, Lim1 (total, weak, and strong staining), Ap2α, or Brn3a double-positive cells in retinas electroporated with *MYCN* at st22/E3.5 and analysed at st34/E8 or st40/E14. **D** Bar graph with fractions of GFP, CC3 double-positive cells in Exp and Ctrl groups electroporated with *MYCN* or GFP control, respectively, at st22/E3.5 and analysed at st40/E14. **E**, **F** Fluorescence micrographs showing GFP, CC3, and Ap2α or Brn3a triple-positive cells in retinas electroporated with *MYCN* at st22/E3.5 and analysed st37/E11 or st35/E9. Mean ± SD, *p < 0.05, **p < 0.01, ***p < 0.001, **A** ANOVA *n*(exp) = 8, *n*(ctrl) = 4, **B** Student’s *t*-test, *n* = 4, **C** Student’s *t*-test, st34/E8, 16 568 cells counted, *n* = 6; st40/E14, 26 855 cells counted, *n* = 6. **D** Student’s *t*-test, 1 357 cells counted, *n* = 4. CC3 cleaved caspase-3, E embryonic day, st Hamburger & Hamilton developmental stage, Vis Visinin. Scale bar in **E** is 25 µm also for **F**.

We electroporated chicken retinas with *MYCN*^T58A^ or c-*MYC*^T58A^ at st22/E3.5, st25/E4.5, or st28/E5.5 and analysed them after 48 h. Only retinas electroporated at st22/E3.5 produced a phenotype of more GFP+Lim1+ cells than control retinas independently of which MYC-variant was used (Figs. 2A and S2A). The expression of Lim1, but not Visinin, can be expected in these early progenitors [36]. To investigate when and how the phenotype observed at st40/E14 arose, we analysed retinas at successive ages from st30/E6.5 to st40/E14, all electroporated at st22/E3.5. Cells double-positive for GFP and Visinin, Lim1, Ap2α, or Brn3a were seen at st30/E6.5 to st35/E9. By st37/E11, the GFP+Brn3a+ cells had disappeared and by st39/E13, GFP+Ap2α+ cells were also gone (Fig. 2B) while the GFP+Visinin+ and GFP+Lim1+ cells remained (Fig. 2C). There were significantly more GFP+ cleaved caspase-3 positive (CC3+) cells in the experimental group than in control (Fig. 2D). GFP+Ap2α+CC3+ and GFP+Brn3a+CC3 were observed at st37/E11 and st35/E9, respectively, also confirmed by TUNEL in *MYCN* retinas (Figs. 2E, F and S2B). This indicated that Brn3a+ and Ap2α+ cells with *MYCN* over-expression are pruned by apoptosis. In contrast, Visinin+ and Lim1_(weak)_+ cells persist despite the *MYCN* expression. Such cell-specific resistance to *MYC*-induced cell death combined with continued, anachronistic proliferation explains the phenotype of accumulating Visinin+ and Lim1+ clustered cells in the experimental group.

### *MYCN*-transformed retinal cells grow in vitro

**Fig. 3:**
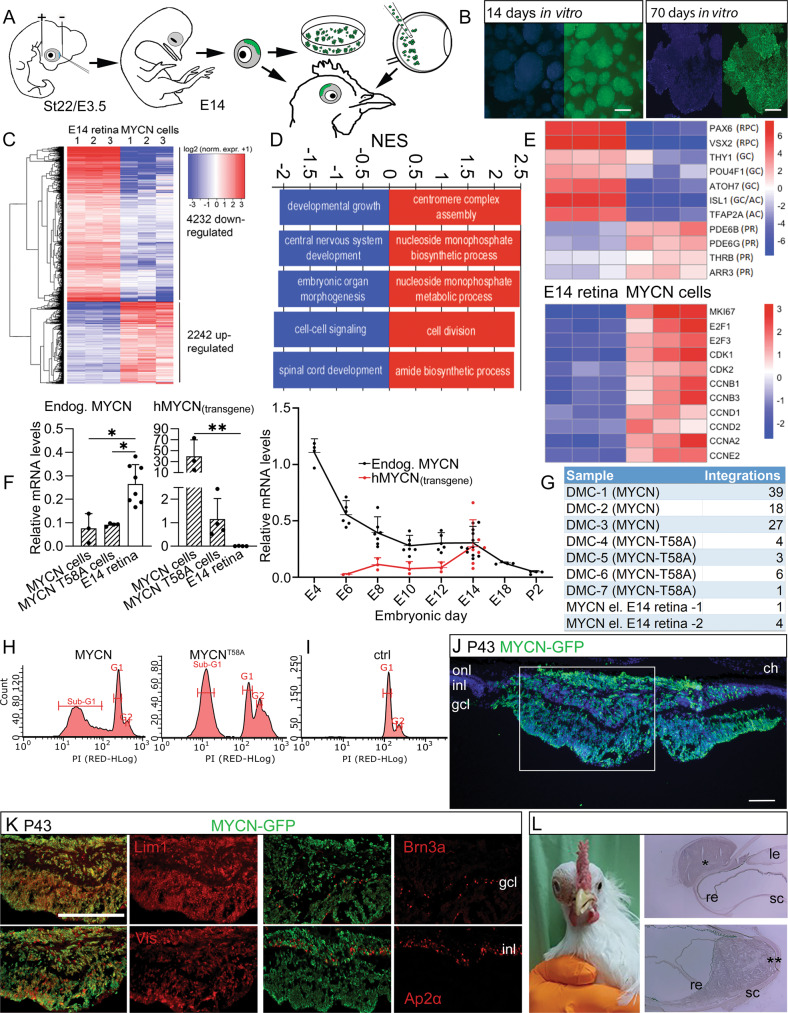
Establishment of cells from *MYCN*/*MYCN*^T58A^ electroporated retina, tumour formation from orthotopically injected *MYCN*^T58A^ cells, and *MYCN* over-expression in adult chicken retina. Cells from electroporated chicken retina were cultured and green-fluorescing, *MYCN*/*MYCN*^T58A^-expressing cells were enriched in vitro, forming pure cultures. The transcriptome of three MYCN cell lines was determined by RNA sequencing and compared to the transcriptome of st40/E14 retina. Differentially expressed genes (DEG) and Gene Ontology (GO) over-representation was determined. *MYCN* expression and the number of vector integrations was quantified and the cell cycle of the established MYCN cells was analysed. Established *MYCN*/*MYCN*^T58A^ cells were orthotopically injected and the eye was analysed. Long-term effects of *MYCN* expression in retina after *in ovo* electroporation was also analysed. **A** Schematic illustration of subretinal plasmid injection and electroporation, establishment of MYC cells, and orthotopic injection. **B** Representative fluorescence micrographs of 2- and 10-week-old (left and right, respectively) primary *MYCN*-cell cultures established from retinas electroporated at st22/E3.5 and dissected at st40/E14. **C** Heat map with log2 normalised expression +1 according to colour scale, featuring DEG (FDR < 0.05, log2 fold-change >1, *n* = 3) in st40/E14 retina and MYCN cells. Of the 13 218 genes that were analysed in the samples, 6 474 were differentially expressed and the majority of them were downregulated. **D** GO gene set enrichment analysis identified 419 GO terms with gene sets of at least 10 genes (FDR < 0.05). The 5 GO terms with the most up- and downregulated DEG based on their normalised enrichment scores (NES) are displayed. Note that the GO terms with downregulated DEG represent neuronal categories and the upregulated ones represent cell-cycle and biogenesis categories related to proliferation. **E** Heat maps with log2 normalised expression +1 according to colour scales, of selected genes for the retinal cell types and for genes regulating cell proliferation. Note that genes related to ganglion (THY1, POU4F1/Brn3a, ATOH7) or amacrine cells (ISL1, TFAP2A/Ap2α) are downregulated and genes related to photoreceptor (PDE6B, THRB, ARR3) functions are upregulated. **F** Relative mRNA levels of endogenous *MYCN* and transgene h*MYCN* depicted in bar graphs with established MYCN and MYCN^T58A^ cells, and ctrl st40/E14 retinas and as an in a line plot of normal and electroporated retinas of ages between st24/E4–P2. **G** Table with number of vector integrations per haploid genome in MYCN and MYCN^T58A^ primary cells and retinas electroporated at st22/E3.5 and analysed at st40/E14. **H**, **I** Cell cycle analysis plots showing number of cells and their DNA content as determined by propidium iodide (PI)-staining of *MYCN* and *MYCN*^T58A^ and control retinal cells. The sub-G1 peak seen in MYCN and MYCN^T58A^ samples are dead/apoptotic cells. **J** Fluorescence micrographs of retina electroporated with a *MYCN* vector at st22/E3.5, stained for GFP and Lim1, Vis, Brn3a, or Ap2α and analysed at P43. Note the absence of ganglion and amacrine cell markers in the tumour. **K** Photograph of a P58 chicken with a large tumour from *MYCN* expression generated by electroporation at st22/E3.5. **L** Bright-field micrograph of haematoxylin staining of extraocular * and intraocular ** tumours. Mean + SD, *p < 0.05, **p *<* 0.01, ***p *<* 0.001; **F** ANOVA, *n* = 3–8. AC Amacrine cells, Ch choroid, ctrl control, E embryonic day, FDR false discovery rate, GC Ganglion cells, gcl ganglion cell layer, inj injection, inl inner nuclear layer, le lens, NES normalised enrichment score, onl outer nuclear layer, PR Photoreceptors, P post-hatch day, re retina, RPC Retinal progenitor cells, sc sclera, st stage. Scale bars in B, 300 µm; in I–M, 10 µm; in N, O, 50 µm.

We dissected and dissociated electroporated regions of st40/E14 chicken retinas from animals electroporated with *MYCN* or *MYCN*^T58A^ and cultured the cells in vitro (Fig. 3A). Approximately 1% of the retinal cells exhibited GFP fluorescence (Fig. S3C). These GFP+ cells proliferated and formed aggregates in suspension (Fig. 3B) whilst GFP negative cells died and were gone after less than two weeks. MYCN and MYCN^T58A^ cells have since then been grown for over 300 days/>30 passages. The transcriptome of three separately established *MYCN* cells cultured for 101 days in vitro was determined by RNA sequencing and compared to that of the st40/E14 retina. Analysis of differentially expressed genes (DEG; FDR < 0.05, log2 fold-change >1, Fig. 3C) and of Gene Ontology gene-set enrichment, showed a clear underrepresentation of the number of transcripts associated with neuronal functions and an overrepresentation of mitosis and biogenesis genes (Fig. 3D). Expression profiles of the MYCN cells were devoid of transcripts associated with GC and AC genes whilst those associated with progenitors for cPRs such as Onecut1, Otx2, and Lhx1/Lim1 [37] as well as other PR genes (Fig. 3E) were found, consistent with the histological analyses. The profiles featured robust expression of cell cycle regulators including cyclins, MKI67/Ki67, E2F1, and −3, and the RNA seq. data of selected genes was confirmed by qRT-PCR (Fig. S3A). Expression levels of the transgene hMYCN were estimated to be two orders of magnitude higher than those of endogenous MYCN with decreasing levels of the endogenous MYCN by st40/E14 (Fig. 3F). The average number of transgene integrations per haploid genome varied (Fig. 3G) and there was no correlation between high expression and a high number of integrations (Fig. S3B).

The cell cycle profiles in MYCN and MYCN^T58A^ cells were distorted compared to normal (Fig. 3H, I). In addition to the G1 and G2 peaks, a conspicuous sub-G1 peak indicative of apoptotic cells, was seen in the MYCN cells but not in the control. LIVE/DEAD assay analysis showed cell viability of 50–75%. The results show that a sufficient number of cells remain viable and proliferate concomitantly with continuous apoptosis in the cultures.

### *MYCN*-transformed retinal cells form tumours in the chicken eye

We orthotopically injected MYCN-cells into st27/E5 embryonic eyes and found that the GFP+Visinin+ and GFP+Lim1+ phenotypes remained at both 1 and 9 days after injection with generation of clusters in the vitreous and on the retina (Fig. S3D).

Eggs windowed at st22/E3.5 have very poor hatchability. Four out of more than 90 electroporated embryos hatched and were considered viable enough for further development. They were euthanised after 43 or 58 days with increased orbital size (Fig. 3P). All four chickens developed tumours in the MYCN-electroporated eye. The cellular phenotype was similar to the embryonic retina with clusters of Visinin+ and Lim1+ cells (Figs. 3J, K and S3D) and the large tumours were histopathologically classified as endo- and exophytic retinoblastoma that infiltrated the optic nerve and penetrated the sclera. The tumours lacked calcification and rosettes but contained necrotic regions.

Eyes from twelve chickens with orthotopically injected MYCN or MYCN^T58A^ cells were studied after 2–6 weeks. All 12 animals had tumorigenic growth in the vitreous, the anterior eye, in the retina, and extra-ocularly (Fig. S3D). The progenitor cell phenotype was confirmed with IR for Visinin, Lim1, and other markers (Fig. S3D).

### *MYCN* expression in human retinal organoids results in tumorigenic growth

**Fig. 4:**
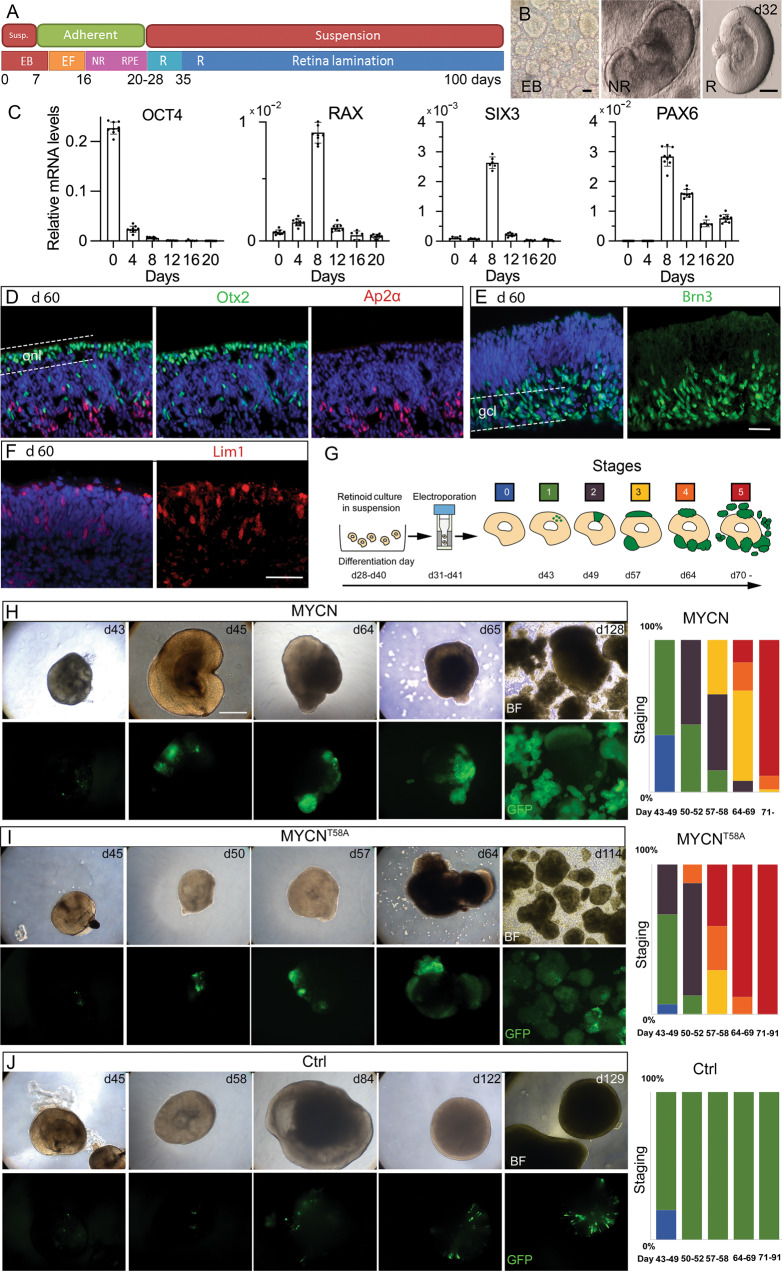
Neoplastic transformation of human retinal organoid cells by over-expression of *MYCN* or *MYCN*^T58A^. Human hESC-derived retinal organoids were generated and electroporated with *MYCN* or *MYCN*^T58A^ piggyBac vectors at a stage when early retinal progenitors were present. The *MYCN* or *MYCN*^T58A^ transgenes were constitutively over-expressed in the organoids, mimicking copy number amplifying *MYCN* oncogenic mutations. The effect of over-expression was monitored by GFP expression from the *MYCN*-GFP bi-cistronic expression units. **A** Schematic illustration of the culture conditions and organoid development. **B** Representative images of organoids during three stages of development. **C** Bar graphs with relative mRNA levels of *OCT4*, *RAX*, *SIX3*, and *PAX6* during early retinal organoid development. Fluorescence micrographs of naïve d60 organoids with IR for **D** Otx2 and Ap2α, **I** Brn3, and **F** Lim1. Dashed lines delineate outer retina/onl or the inner retina/gcl. **G** Schematic of the electroporation protocol for transfecting developing organoids. The image also depicts the scores (0-5) of the neoplastic stages and their associated phenotypes. **H**–**J** Brightfield and fluorescence low magnification micrographs of the progressive growth of GFP-positive cell populations with representative examples of organoids with tumorigenic neoplastic growth in *MYCN* transformed organoids (green fluorescence) as well as stacked histograms showing the percentiles of organoids belonging to each neoplastic stage over time in **H** *MYCN*-, **I** *MYCN*^T58A^-, and **J** control-electroporated organoids. BF brightfield, d days in culture (organoid age), EB embryoid body, EF eye field, gcl ganglion cell layer, IR immunoreactivity, NR neural retina, onl outer nuclear layer, R retina, RPE retinal pigment epithelium. Scale bars in **B** are 200 µm, in **E** and **F** 50 µm, and in **H** 200 µm.

Our next step was to investigate whether the findings in chicken were also valid for human by exploring the effects of MYCN-over-expression in hESC-derived organoids. Retinal organoids were established [38] (Fig. 4A, B) and the formation of eye-field and neural retina was monitored by qRT-PCR analysis of *OCT4, RAX, SIX3*, and *PAX6* mRNA [38] (Fig. 4C). The formation of cell types is conserved with GCs, cPRs, HCs, and ACs forming early [39, 40]. We used a Brn3 antibody that recognises Brn3a, -b, and -c for GCs, Otx2 instead of Visinin for the PR lineage, Lim1 for HC/cPR progenitors, and Ap2α for ACs. Organoids were cultured for more than 150 days and their development recapitulated previously published results (Figs. 4D–F and S4A) [41, 42].

Organoids were electroporated with MYCN-GFP piggyBac vectors on days 38–41 (Fig. 4G). Electroporated retinal organoids recovered their optic cup structures after 18–24 h. Organoids with a normal structure were studied and, typically, >75% of transfections were successful. Four experimental groups were compared: (1) *MYCN*, (2) *MYCN*^T58A^, (3) GFP control, and (4) a naïve non-electroporated control group. The GFP+ region expanded robustly in the *MYCN* and *MYCN*^T58A^ groups. By 3–4 weeks, GFP+ cells detached from the organoids and formed non-adherent GFP+ cell aggregates. The GFP control group was normal (Fig. 4H–J). The organoids were scored in five stages based on the size and structure of the GFP+ regions (Fig. 4G). 89% of organoids in the *MYCN* group and 100% in the *MYCN*^T58A^ group reached stage 5 within 7 weeks whilst none in the GFP control group advanced past stage 1 (Figs. 4H–J and S4B). The controls had normal cell and retinal organoid morphologies, even after extended periods in culture (>150 days). Age-matched naïve organoids had normal morphologies. Expression of *MYCN* or *MYCN*^T58A^ in developing organoid cells led to neoplastic growth of transformed cells.

### MYCN over-expressing human retinal organoid cells express markers of PR progenitors

**Fig. 5:**
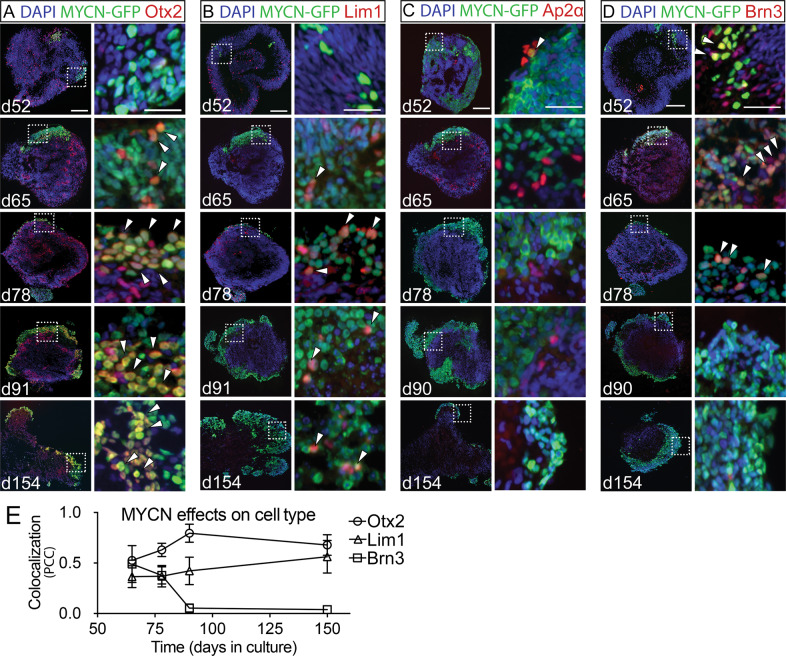
Cell-specific survival following over-expression of *MYCN* in human retinal organoids. Immunohistochemical analysis of human retinal organoids electroporated on d39–41 with a bi-cistronic *MYCN*-GFP piggyBac expression vector. The electroporation generated stable and robust *MYCN* over-expression in all cell types driven by the CAG promoter and visualised by GFP. Time-points between d52 and 154 were analysed. Electroporation at d39–41 targets retinal progenitors before neuronal differentiation. Fluorescence micrographs of MYCN organoids of various ages are shown with IR for GFP and **A** Otx2, **B** Lim1, **C** Ap2α, and **D** Brn3. Dashed line-boxes in the left panels indicate magnified regions shown in the right panel images. White arrowheads depict examples of colocalization of *MYCN*-GFP with either of the retinal cell-markers. **E** Line graph showing colocalization of *MYCN*-GFP with Otx2, Lim1, or Brn3 IR. Colocalization is illustrated by Pearson’s correlation coefficient (PCC) where 1 is high correlation (colocalization) and 0 is low. Mean ± SD, n = 4. d, days in culture (organoid age). Scale bars in left image panels are 100 µm and in right panels are 25 µm.

GFP+ cells on days 52–65 organoids from experimental groups 1–3 were IR for Otx2, Lim1, Ap2α, or Brn3 IR (Figs. 5A–D and S5A, B). From day 78 onwards, Otx2 and Lim1 IR dominated the GFP+ regions of the *MYCN* and *MYCN*^T58A^ organoids. As the number of GFP+Otx2+ and GFP+Lim1+ cells increased and GFP+Brn3+ and GFP+Ap2α+cells decreased after day 78 (Figs. 5E and S5A). This change was associated with increased size and altered morphology of the GFP+ cell clusters. No accumulation of GFP+ cells was seen in controls. These results show that GFP+ cells expressing Otx2 and/or Lim1 expand and accumulate, unlike those expressing Brn3 or Ap2α, which instead were absent from the *MYCN* and *MYCN*^T58A^-transfected organoids at day 90 (Figs. 5E and S5A, S5B). It is worth noting that while GFP+ AP2α+ cells were not present in the day 150 organoids, a few organoids had GFP+ Brn3+ cells that re-appeared (Figs. 5D and S5B). These cells were conspicuous but few and did not change the results of the day 150 co-localisation analysis (Fig. 5E).

### MYCN-transformed human retinal organoid cells proliferate and remain less differentiated in the PR/HC lineage

**Fig. 6:**
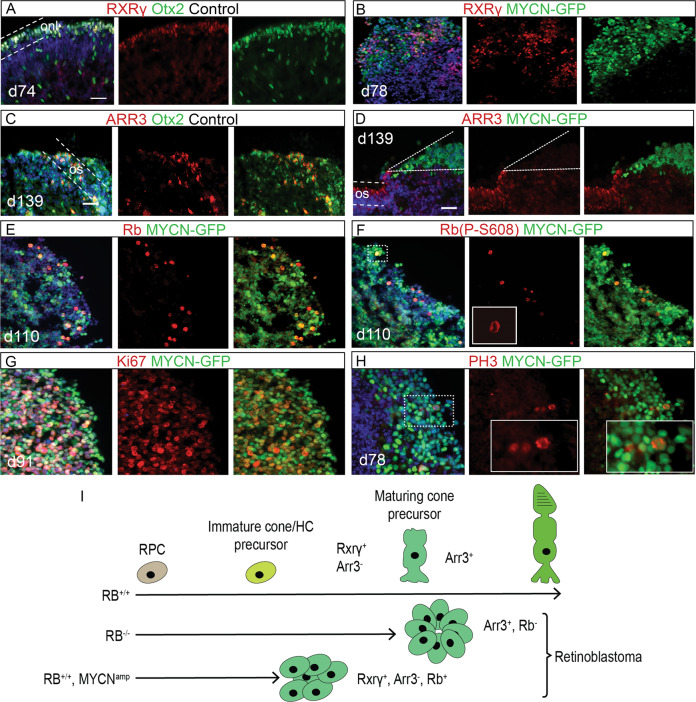
Effects of *MYCN* over-expression on differentiation and proliferation in human retinal organoids. Immunohistochemical analysis of human retinal organoids electroporated on d39–41 with a bi-cistronic *MYCN*-GFP piggyBac expression vector. The electroporation generated stable and robust *MYCN* over-expression in all cell types driven by the CAG promoter and visualised by GFP. Fluorescence micrographs of **A** and **C** naïve control organoids and **B**, **D**–**H** *MYCN*-electroporated organoids aged between d74–139 in culture. IR for **A** RXRγ and Otx2; dashed lines delineate onl, **B** GFP and RXRγ, **C** ARR3 and Otx2; dashed lines delineate the outer segments (os) **D** GFP and ARR3; note the absence of colocalization between ARR3 positive cells and GFP positive cells delineated with the dashed lines, **E** GFP and total Rb, **F** GFP and S608-phosphorylated Rb, **G** GFP and Ki67 (proliferation antigen Ki67), and **H** GFP and PH3 (phospho-histone 3). Dashed line-boxes indicate magnified region in right panels. Note the GFP, PH3 double-positive mitotic figure in anaphase. **I** Schematic illustration depicting effects of bi-allelic loss of *RB1* or *MYCN* over-expression on retinal progenitor cells. Homozygous loss of *RB1* function generates differentiated tumour cells that express ARR3 while over-expression of *MYCN* generates less differentiated cells that do not express ARR3. d days in culture (organoid age), IR immunoreactivity, onl outer nuclear layer, os outer segments. Scale bars are 50 µm.

Even after 150 days in culture, MYCN-GFP+ cells remained with round somas and a phenotype of predominantly Otx2 and/or Lim1 IR. The retinoid acid X receptor-γ (RXRγ) is expressed during early cone differentiation [43], and Arrestin 3 (ARR3) in maturing cones [44]. In the naïve control group, RXRγ + organoid cells arose prior to ARR3+ cells, which were also Otx2+ (Figs. 6A, C and S6A). GFP+RXRγ+ cells were seen after day 78 in MYCN retinal organoids (Fig. 6D). GFP negative, ARR+ cells but not GFP+ARR3+ cells could be found (Figs. 6D and S6A), indicating that *MYCN*-expressing cells do not differentiate to the same extent as normal organoid cells do.

Day 65 to 110 organoids were analysed using IHC for the proliferation markers Rb, Ki67, and PH3 [45, 46]. Both total Rb and S608-phosphorylated Rb, (Rb P-S608; phosphorylated in late G2- and M-phase [47]) were analysed. The number of GFP+Rb+ cells increased in MYCN-organoids and Rb (P-S608) IR was predominantly over mitotic figures (Figs. 6E, F and S6B). A majority of the GFP+ day 65–111 MYCN-organoid cells were Ki67+, and PH3+ cells with mitotic figures were also present in these models (Figs. 6G, H and S6B). Taken together, the *MYCN*- and *MYCN*^T58A^-expressing organoid cells exhibit an undifferentiated, ARR3-negative, and proliferating phenotype. It is worth noting that cells in *RB1*^−/−^ organoids express ARR3 [20, 21] (Fig. 6I). The Otx2+, RXRγ+, ARR3-negative phenotype in retinal organoids expressing *MYCN* is indicative of less differentiated cells.

## Discussion

In this work, we have studied the effects of *MYCN* over-expression in developing retinas. Our results show that MYCN acts as an oncogene in *RB1*-proficient retinas, leading to neoplasia in two novel models of retinoblastoma: one with *MYCN* over-expression in the chicken retina and one in hESC-derived retinal organoids. MYCN^A^ gene copy-number mutations causing over-expression is one of the most frequent mutations found in retinoblastoma after *RB1* loss-of-function [22]. The *RB1* and MYCN^A^ mutations often co-occur, though tumours with MYCN^A^ without *RB1* loss-of-function have been described [25]. The *MYC*-family members and their extended network of proteins have pleiotropic functions, regulating cell growth, proliferation, differentiation, and apoptosis. Though their expression is restricted to specific developmental stages and tissues, their proteins have similar functions. Oncogenic mutations often produce higher levels of MYC protein or increase its stability such as gene amplifications or the T58A mutation [28]. Aberrant, increased expression of *MYCN* is also associated with other paediatric cancers such as neuroblastoma and medulloblastoma [48, 49]. The MYCN expression levels in this study (Fig. 3C and E) were of the same magnitude as seen in neuroblastomas [48]. Development of the tumorigenic phenotype was concomitant with the gradual decrease of endogenous *MYCN* and sustained transgene-*MYCN* expression in the chicken retina (Fig. 3E). A similar decrease of endogenous *MYCN* mRNA levels is seen in human retina [6].

The neoplastic effects of wild-type and T58A variants of human *MYCN* and c-*MYC* in the chicken retina were similar, consistent with different MYC-proteins having partly redundant activities. By using the ubiquitous CAG promotor, the stage- and tissue-specificity of MYC expression was over-ridden, providing an explanation for how c-*MYC* could drive the phenotype despite *c-MYC* mutations not being common in retinoblastoma [23, 25, 50]. We do not rule out the possibility that over-expression of *MYCN* or c-*MYC* could produce different phenotypes, but the results of this study demonstrate that they both can trigger tumorigenic growth of retinal Visinin+ and Lim1+ cells, yielding similar results.

Recent work based on a multi-omics data analysis of 102 enucleated retinoblastomas proposed a subdivision into two types [51]. Type 1 is associated with few genetic aberrations other than *RB1* mutations and with phenotypic properties of immature cones. Type 2 retinoblastoma frequently harbours more genetic aberrations, including MYCN^A^, and is associated with markers of less differentiated cones, retinal progenitors, and retinal GCs. Type 2 is also more aggressive, has a higher propensity for metastasis, and is typically diagnosed in younger children, compared to type 1 retinoblastoma [51]. The tumour features presented here, with less differentiated cPR/HC progenitors, are akin to those of type 2 retinoblastoma. Though markers for undifferentiated cPRs and not for GCs (Brn3) were clearly predominant among the GFP+ cells in the MYCN-transformed organoids, GFP+Brn3+ cells in the day 150 organoids were found. Brn3 transcription factors (POU4F1–3) are essential in the early GC differentiation programme and those few cells in the old organoids may indicate a potential for re-initiation of the GC differentiation programme. This finding is consistent with that of type 2 tumours that present neuronal progenitor and GC markers [51], and similar features have been found in old (>90 days) *RB1*-deficient retinal organoids, derived from either hESCs or patient iPSCs [20, 21, 51]. The dominating phenotype, however, is that of cone photoreceptors of various degrees of maturation or stress [20, 21, 51]. We did not find GC markers in the chicken MYCN-driven tumour cells but do not rule out the possibility that the MYCN-resistant progenitors may have or subsequently develop such properties. It is worth noting that the dominating phenotype in the lineage of PRs, in the chicken retina and human retinal organoid MYCN-transformed cells in the present study, is comparable to that of *RB1*-deficient organoids [20, 21, 51]. This is consistent with the cell of origin being similar for *RB1*-deficient and *RB1*-proficient, MYCN-induced neoplasms.

Our results indicate that *MYCN* over-expression is sufficient in inducing tumorigenic growth when occurring in the fate-restricted cPR/HC progenitor in the chicken retina and that the growth develops into less differentiated retinoblastoma. The tumorigenic phenotype was fully penetrant after successful over-expression in the chicken retina in vivo and in organoids. The phenotype being less differentiated is based on (i) the immature cell morphologies and expression profile, (ii) the cellular positions and lack of rosettes, and iii) the active proliferation, both in chicken retina and in organoids. The expression of a large set of mitosis genes, including *E2F1*, *E2F3*, and *E2F8* were robustly upregulated in the MYCN cells compared to E14 retina. Myc and E2Fs synergistically regulate the cell cycle in a positive feedback loop wherein *E2F1* and *E2F3* are targets for Myc-regulation [52]. Over expression of MYCN in the context of inactivated *RB1* increased the expression of E2Fs and triggered neoplastic growth in retina [26]. MYCN-induced high levels of E2F expression in these cells is likely to override proficient *RB1*gene cell regulatory functions. Furthermore, the increased phosphorylation of Rb seen in primary MYCN^A^ retinoblastoma [27] may aggravate the dysregulation and contribute to neoplasia. A similar and increased expression of both photoreceptor and E2F cell cycle regulatory genes are seen in several *RB1* mutant retinoblastoma cell lines with MYCN^A^ [53].

Our findings that h*MYCN* transforms *RB1*-proficient retinal cells with high frequency differ from those of the studies of h*MYCN* over-expression in mouse in which tumorigenic growth with high frequency only occurred in *Rb1*^−/−^ or *Rb1*^−/−^*Rbl2*^−/−,^ mice [26]. The expression profiles had both increased expression of E2F- and ribosome biosynthesis-related gene sets, excessive proliferation, and led to tumorigenic growth with anaplastic features. Recent work also showed that MYCN as an oncogene in retinoblastoma modulates several systems that contribute to enhanced viability and invasiveness [54]. The mouse MYCN-induced *Rb1*^−/−^ cells were not described as expressing photoreceptor genes [26] in accordance with several mouse retinoblastoma models, including the *Rb1*^−^^/−^*Rbl2*^−^^/−^ mice that do not express specific cPR profiles but rather profiles with ACs and HCs, suggesting an alternative cell of origin [55, 56]. This discrepancy may be associated with species-specific neurogenesis and cell type-composition of cPR/HCs in mouse compared to chicken and possibly also to human [12]; the mouse retina is rod-dominant with only 1–3% of the PRs being cones and mice have one HC-type (H1, B-type), compared to chicken which has a cone-dominant retina and three HC subtypes (H1–3 or A, B, C-types) [12, 57]. The discrepancy may also be related to the intrinsic properties of the fate-restricted cPR/HC progenitor [7, 9, 37] identified in chicken retina but has yet to be identified in the rodent retina. Consistent with the requirement of co-occurring *Mycn* and *Rb1* mutations to induce tumorigenic growth in mice, knock-out of only *Rb1* is not sufficient for tumorigenesis [58–60], a phenomenon not seen in human retina or retinal organoids [6, 61]. Targeted deletion of *RB1* in hESCs gives transformation with high frequency [20]. Tumorigenic growth in mouse is seen after knockout of 2.5 × *Rb* paralogs [11] or when combined with other tumour suppressor gene deficiencies [62].

Stage-restricted retinal tumorigenesis has been described in human but not mouse [44]. In chicken, we found restrictions to before st27/E5 (Fig. 2A). This age is before the terminal mitoses of HC progenitors, which are neither sensitive to DNA breaks nor activation of the DNA damage response system [16, 18]. This age also coincides with the formation of the restricted cPR- and HC-progenitors from multipotent retinal progenitors [63]. Moreover, the restricted period of naturally-occurring cell death, during which pruning of *MYCN*-transformed cell-types occurs, defines the stage of the tumorigenic events. A similar process has been described in the human retina [3] and is inferred to occur in the organoids. This cell-loss is distributed over several weeks in the organoids compared to 36 h in the chick, making these studies in the chick embryo more feasible.

In conclusion, our data demonstrate that *MYC* over-expression is sufficient to initiate tumorigenic growth with high frequency in the presence of normal *RB1* functions. *MYCN* over-expression transforms retinal progenitor cells for cPRs and HCs, implicating the progenitors as the cell-of-origin for this subtype of *MYC*-induced retinoblastoma in chicken retina and in human retinal organoids. Our results also imply that the cell-specificity for *MYC*-induced tumorigenesis is a result of selective survival of cells in the cPR/HC lineage, which tolerate the MYCN expression and undergo carcinogenesis. These cells proliferate beyond the point of normal terminal mitosis without differentiating, instead maintaining progenitor features and form undifferentiated cell clusters that develop into metastatic and anaplastic retinoblastoma within 7–9 weeks in young chickens. We present a new in vivo model of retinoblastoma with MYCN^A^ and wild type *RB1* in the chicken embryonic retina that recapitulates the early events that lead to malignant transformation. This model can serve as an hypotheses-generating tool regarding early events in carcinogenesis, which can then be tested in the human retinal organoid system. The tumour modelling in this work may also be useful for testing novel and specific drugs targeting retinoblastoma with MYCN^A^ mutations.

## Material and methods

For additional and detailed information see Supplementary materials and methods.

### Animals and human stem cells

Fertilised White Leghorn eggs (*Gallus gallus*; Ova Production AB, Vittinge, Sweden), were incubated at 37 °C in a humidified incubator (8204/MP, Grumbach, Asslar, Germany). Embryonic age was determined by staging (st) according to Hamburger and Hamilton [64] or by embryonic days (E). Animal experiments were carried out in compliance with the guidelines set by the Association for Research in Vision and Ophthalmology and were approved by the Regional animal ethics committee in Uppsala, Sweden (Dnr C90/16, C159/15, 5.8.18-09718/2021).

### Human ESC and differentiation of retinal organoids

Organoids were obtained following the protocol by Zhong et al. 2014 [38] with minor modifications. We used HS980 ESCs [65] grown on human recombinant laminin. The work was performed in accordance with the Declaration of Helsinki and approved by the Swedish Ethical Review Authority in Uppsala (Ethical permit number EPN-20117745-31/3). For ESC culturing and the differentiation protocol, see Supplementary Materials and Methods.

### DNA Constructs

Information regarding vectors and their experimental usage is listed in Table S1.

### Electroporations

The developmental stage of fertilised eggs was estimated by candling, and the exact Hamburger-Hamilton stage was verified after windowing the egg to enable injections and electroporation of the embryo. St22/E3.5-st28/E5.5 chicken eyes were electroporated in ovo after subretinal injection of plasmid vectors containing c-*MYC*/*MYCN*, their T58A-variants, or GFP-control. Retinal organoids were electroporated in 1 mm cuvettes (732-0020, VWR, Radnor, PA, USA).

### Establishment of in vitro MYC cultures and orthotopic injections

Following in ovo electroporation at st22/E3.5 with GFP-expression vectors, the green-fluorescing region of st40/E14 embryos was dissected. The MYCN/MYCN^T58A^-GFP expressing cells were dissociated and seeded onto a 35 mm dish containing RPMI1640 (21875034, Gibco) supplemented with 10% FBS (16000044, Gibco), 1% NEAA (11140035, Gibco) and 1% pen:strep (15140122, Gibco). After 7 days in vitro, cells in suspension were transferred to a 100 mm dish (83.3902, Sarstedt, Nümbrecht, Germany). Medium was changed twice weekly. For orthotopic injection, cells were resuspended and dissociated in 1× PBS+/+ and mixed with Fast Green (F7252, Sigma-Aldrich) to aid in visualisation. After windowing of st44/E18 eggs, approximately 20,000 cells in 0.2 µl were injected subretinally or intraocularly into the right eye.

### Flow cytometry and cell cycle analysis

St34/E8 and st40/E14 chicken retinas electroporated at st22/E3.5 with the pB-CAG-MYCN-IRES-GFP vector as well as established *MYCN* expressing cells were analysed using a Guava easyCyte 8 Flow Cytometer (Luminex, Austin, TX, USA).

### Quantitative reverse transcriptase PCR analysis

Total RNA from dissected retina and cultured cells was extracted with TRIzol. In brief, cDNA was subjected to qRT-PCR using IQ™ SYBR® green Supermix (1708882, Bio-Rad Laboratories AB, Hercules, CA, USA) and the Ct values were normalised to β-actin and TATA box-binding protein (TBP). See Table S2 for primers.

### Analysis of piggyBac integrations per haploid genome

Quantitative PCR analysis with primers (Table S2) against the piggyBac transposon element and two single-copy reference genes, which provided a relative baseline as one copy per haploid genome [66], were used to calculate the number of construct integrations after in ovo electroporation. Genomic DNA was serially diluted and each dilution was subjected to qPCR analysis.

### Immunochemistry

Dissected chicken eyes and retinal organoids were fixed in PFA, embedded, and sectioned on a cryotome. Sections were rehydrated in 1× PBS and incubated in primary antibody solution overnight. For antibodies and dilutions see Table S3.

### Microscopy, image, and data analysis

Fluorescence micrographs were captured using a Zeiss Axioplan 2 or a Zeiss Imager Z2 microscope (Carl Zeiss Microscopy GmbH, Jena, Germany). Cell counting was manual with assistance of the software Fiji-ImageJ2. Colocalization was analysed by use of CellProfiler. Contrast of fluorescence images was enhanced at the microscope using the Zeiss capture and image analysis software (Axiovision or ZEN). Considerations for experimental design and determination of sample sizes see supplementary methods. At least four histological sections per animal from four animals (biological replicates) for each time point were analysed and used for cell counting unless otherwise stated in figure legends. Distribution is presented as mean and SD. Data were analysed with one-way ANOVA followed by Tukey’s multiple comparison post-hoc test or Student’s *t* test using GraphPad Prism (GraphPad Software Inc. San Diego, CA, USA), and statistical significance was set to *p* < 0.05. Statistical analysis and total number of cell counts for each comparison are also presented in the figure legends.

### RNA sequencing

Total RNA from three samples each of MYCN cells and st40/E14 retina was extracted with Qiagen RNeasy Micro Kit (#74004, Qiagen, Hilden Germany) following manufacturer’s instructions. The library establishment and RNA sequencing were performed by SNP&SEQ Platform of National Genomics Infrastructure in Sweden. The library was prepared using the TruSeq Stranded mRNA Library Preparation Kit with polyA selection (Illumina Inc., San Diego, CA, USA). Sequencing was performed on SP Flow Cell using the NovaSeq 6000 system and v1.5 sequencing reagents (Illumina Inc.).

### Analysis of RNA sequencing data

Quality check and alignment of the data was done by using the nf-core/rnaseq pipeline (v3.4) [67] with adjustments to the alignment parameters. To extract fragment counts, featureCounts (v2.0.0) [68] was used. The differential expression analysis was done by edgeR with FDR < 0.05 and log2 fold-change >1. GO gene-set enrichment analysis was done by using gseGO [69]. Terms with at least 10 genes were used for downstream analysis and visualisation. All analyses were performed using R Statistical Software (v4.1.1) [70]. Additional information regarding alignment parameters and analysis in Supplementary Material. The seq data will be available under accession GSE199162 in the GEO data repository.

## Supplementary information

Supplementary material

Supplementary figure S1

Supplementary figure S2A

Supplementary figure S2B

Supplementary figure S3A

Supplementary figure S3B

Supplementary figure S3C

Supplementary figure S3D

Supplementary figure S4A

Supplementary figure S4B

Supplementary figure S5A

Supplementary figure S5B

Supplementary figure S6A

Supplementary figure S6B

## Data Availability

All data generated or analysed during this study are included in the published article and its supplementary information files. Raw data is available under accession GSE199162 in the NCBI GEO data repository or from the corresponding author upon reasonable request.
