# Supplementary material for: MYCN induces cell-specific tumorigenic growth in RB1-proficient human retinal organoid and chicken retina models of retinoblastoma
Source: Oncogenesis. 2022 Jun 21;11(1):34. doi: 10.1038/s41389-022-00409-3 (PMC9213451; doi:10.1038/s41389-022-00409-3)
Supplement: Supplementary file 1 — Supplementary material [file 41389_2022_409_MOESM1_ESM.docx]

# Supplementary material

*MYCN* induces cell-specific tumorigenic growth in *RB1*-proficient human retinal organoid and chicken retina models of retinoblastoma

Maria K E Blixt, Minas Hellsand, Dardan Konjusha, Hanzhao Zhang, Sonya Stenfelt, Mikael Åkesson, Nima Rafati, Tatsiana Tararuk, Gustav Stålhammar, Charlotta All-Eriksson, Henrik Ring, and Finn Hallböök.

**Supplementary Table S1:** Schematic diagrams of vector constructs with functional elements and sizes.

MYCN and GFP are separated by an IRES and are expressed as a bi-cistronic transcription unit. CAG; Chicken β-actin promoter with a CMV enhancer^1^, cyt; cytoplasmic localization, GFP; Green Fluorescent protein, HC; horizontal cell, IRES/IRES2; Internal ribosome entry site, LSL; loxP-STOP-loxP-CRE (removed via Cre-recombination), nuc; nuclear localization, pB; piggyBac vector, RXRγ208; 208 bp regulatory element from the RXRγ gene that drives expression in cone and horizontal cell progenitors^2^.

| Vector | Description | Featured in Figure(s) |
| --- | --- | --- |
| PBase (3 761 bp) | PiggyBac “helper vector”, encodes the transposase PBase for genomic integration of the pB-vectors. | All experiments that require piggyBac integration. |
| 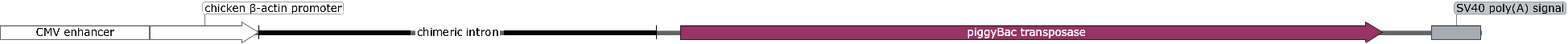 | | |
| pB-CAG-GFP (5 802 bp) | PiggyBac-integrating, CAG-driven GFP^nuc^ control vector. | S1c |
| _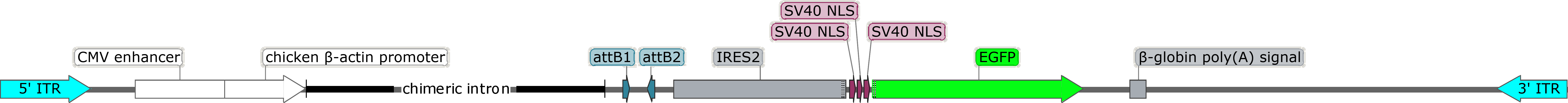_ | | |
| pB-CAG-LSL-GFP (5 929 bp) | PiggyBac-integrating, CAG-driven GFP^cyt^ control vector. An LSL sequence upstream of *GFP*. | 1D, 1I, 4J, S1e |
| 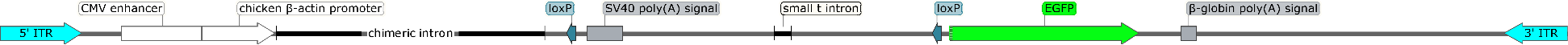 | | |
| pCMV-CRE (4 300 bp) | Transient, ubiquitous Cre expression vector. | 1D, 1I, 4J |
| 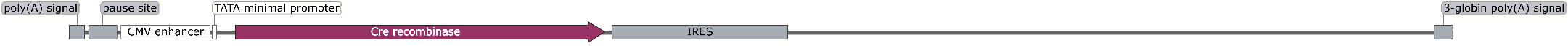 | | |
| pRXRγ208-CRE (4 381 bp) | Cre expression under control of the 208 bp RXRγ regulatory element; drives expression in progenitors of cone PRs and HCs. | S1d-e |
| 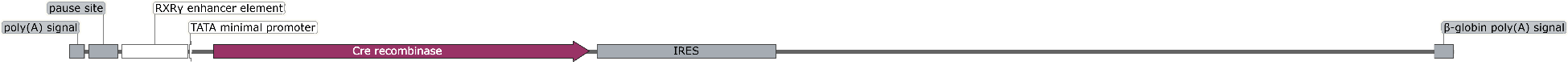 | | |
| pB-CAG-MYCN^T58A^-IRES-GFP  (6 732 bp) | PiggyBac-integrating, CAG-driven MYCN^T58A^ expression vector with GFP^nuc^ in a bi-cistronic transcription unit. | S5, S1b, S1f |
| 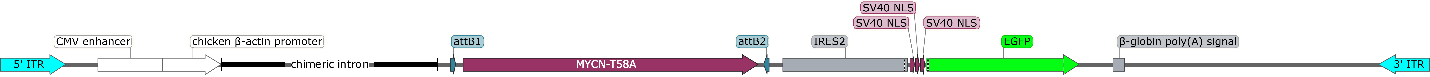 | | |
| pB-CAG-LSL-MYCN^T58A^-IRES-GFP  (7 935 bp) | PiggyBac-integrating CAG-driven MYCN^T58A^ expression vector with GFP^nuc^ in a bi-cistronic transcription unit. An LSL sequence upstream of the transcription unit. | S2d |
| 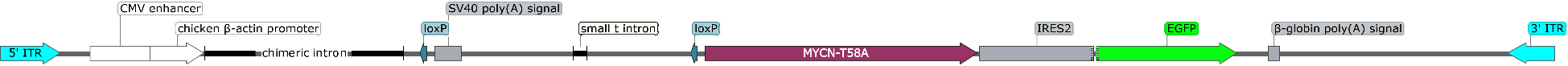 | | |
| pB-CAG-MYCN-IRES-GFP  (6 732 bp) | PiggyBac-integrating, CAG-driven MYCN expression vector with GFP^nuc^ in a bi-cistronic transcription unit. | 1B,1E-H, 1J, 1L-Q, 2C, 2E-F, 3C, 3E, 3G, 3N-P, 4H, 5A-E, 6B, 6D-H, S1a, S6Ab, S6Ad, S6B |
| 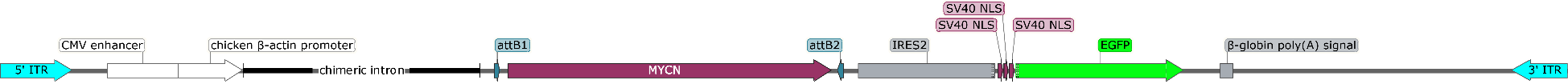 | | |
| pB-CAG-cMYC^T58A^-IRES-GFP  (6 602 bp) | PiggyBac-integrating, CAG-driven c-MYC^T58A^ expression vector with GFP^nuc^ in a bi-cistronic transcription unit. | S1c |
| 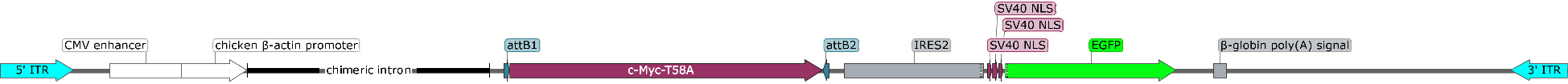 | | |
| pCAG-MYCN (CIG-DV) (4 909 bp) | Transient, CAG-driven MYCN expression vector with GFP^nuc^. | 2A |
| 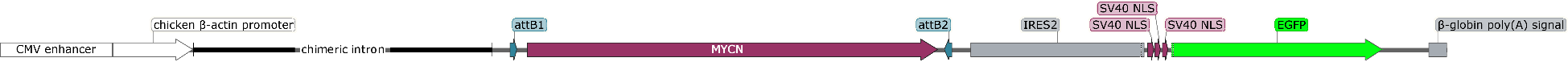 | | |
| pCAG-MYCN^T58A^ (CIG-DV) (4 909 bp) | Transient, CAG-driven MYCN^T58A^ expression vector with GFP^nuc^. | 2A |
| 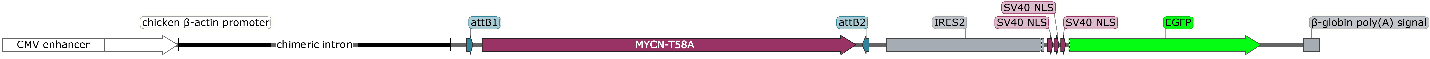 | | |
| pCAG-c-MYC^T58A^ (CIG-DV) (4 779 bp) | Transient, CAG-driven c-MYC^T58A^ expression vector with GFP^nuc^. | 2A |
| 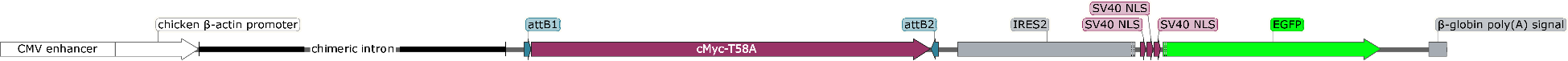 | | |
| pCAG-GFP (pZG) (2 691 bp) | Transient, CAG-driven control GFP^cyt^ expression vector. | 2J |
| 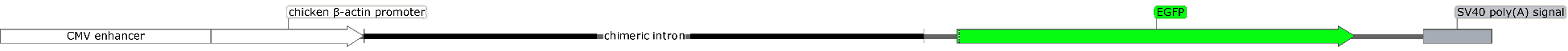 | | |

**Supplementary Table S2**: List of primers for qRT-PCR and q-PCR used in this study

| **Gene** | **Forward 5'-3'** | **Reverse 5'-3'** | **Source** |
| --- | --- | --- | --- |
| β-actin | aggtcatcaccattggcaatg | cccaagaaagatggctggaa | PMID:25483080 |
| GAPDH | cctgacctgccgtctagaaaaa | tgtcgctgttgaagtcagagga |  |
| GFP | acgtaaacggccacaagttc | aagtcgtgctgcttcatgtg | from vector |
| Ki67 | tggtgatttgttacctcagcct | gtggcgagtttccaaatgg | XM_015289038.1 |
| MYCN (Chicken) | cccgtccagtaaaacccatga | tggaacgtggtcccttaacgt |  |
| MYCN (Human) | ttgtacaaaaaagcaggctccg | agcgagtcaaactcgaggtctg | from vector |
| OCT4 | ggagaatttgttcctgcagtgc | agaaccacactcggaccacatc |  |
| PAX6 | ccattggctgactgttcatgtg | cctcctcatactcctgcatgct |  |
| piggyBac 5' ITR | aatccgtcgctgtgcatttag | tttgactcacgcggtcgtt |  |
| PMCH | cgaaatggagacggaactgaa | catccaagaagctttcctcaatct | PMID: 19604406 |
| POMC | gctacggcggcttcatga | cgatggcgtttttgaacagag | PMID: 19604406 |
| RAX | gaccctggcatgacgcaatat | cgtggtcatccttttcccaagt |  |
| SIX3 | caggtcagtccatggtattccg | tactcgccagaagtatggagcg |  |
| TBP | tagcccgatgatgccgtat | gttccctgtgtcgcttgc | PMID:25483080 |

**Supplementary Table S3**: List of antibodies used in this study.

| **Antibody** | **Host** | **Dilution** | **Company** | **Catalogue number** |
| --- | --- | --- | --- | --- |
| Ap2α | mouse | 1:200 | DSHB* | 3B5 |
| ARR3 | rabbit | 1:1000 | Invitrogen | PA5-75301 |
| Brn3, pan | goat | 1:1000 | Santa Cruz Biotechnology | sc-6026 |
| Brn3a | mouse | 1:200 | Merck Millipore | MAB1585 |
| Caspase-3, cleaved | rabbit | 1:1000-4000 | Cell Signalling Technology | #9661 |
| GFP | rabbit | 1:4000 | Abcam | ab290 |
| GFP | goat | 1:4000 | Abcam | ab5450 |
| Isl1 | mouse | 1:200 | DSHB* | 40.2D6 |
| Isl2 | mouse | 1:200 | DSHB* | 51.4H9 |
| Ki67 | rabbit | 1:1000 | Abcam | ab15580 |
| Lim1/2 | mouse | 1:20 | DSHB* | 4F2-s |
| Lim3 | mouse | 1:200 | DSHB* | 67.4E12 |
| Onecut1 | rabbit | 1:200 | Santa Cruz Biotechnology | sc-13050 |
| Onecut2 | sheep | 1:500 | R&D Systems | AF6294 |
| Otx2 | goat | 1:500 | R&D Systems | AF1979 |
| Pax6 | mouse | 1:200 | DSHB* | Pax6 |
| PH3 | goat | 1:400 | Santa Cruz Biotechnology | sc-12927 |
| Prox1 | rabbit | 1:4000 | Chemicon | AB5475 |
| Rb | rabbit | 1:1000 | Abcam | ab39690 |
| Rb (P-ser608) | rabbit | 1:1000 | Abcam | ab60025 |
| RXR- γ | mouse | 1:1000 | Santa Cruz Biotechnology | sc-365252 |
| Visinin | mouse | 1:1000 | DSHB* | 7G4 |
| *DSHB; Developmental Studies Hybridoma Bank | | | | |

# Supplementary material and methods

*MYCN* induces cell-specific tumorigenic growth in *RB1*-proficient human retinal organoid and chicken retina models of retinoblastoma.

Maria K E Blixt*, Minas Hellsand*, Dardan Konjusha*, Hanzhao Zhang, Sonya Stenfelt, Mikael Åkesson, Nima Rafati, Tatsiana Tararuk, Gustav Stålhammar, Charlotta All-Eriksson, Henrik Ring, and Finn Hallböök.

## DNA constructs

PiggyBac integration vectors with a chicken β-actin gene (CAG) promoter were used to generate stable high-level expression in chicken retina and human retinoids. To generate stable expression in progenitors for PR and HCs, a Cre and transcriptional stop flanked by loxP sequences system was used. In the presence of Cre protein the stop was removed to release expression from CAG promoter. *CRE* expression was driven by a 208 bp RXRγ gene regulatory sequence^2^. The *MYC* expression vectors have a bi-cistronic transcription unit with *MYC* sequence, an internal ribosome entry site, and GFP with nuclear or cytoplasmic localization. Control vectors were without *MYC* sequences. See supplementary Table S1.

## Electroporations

After eggs had been incubated until their presumed developmental stage, they were candled to estimate the developmental stage. After opening the egg, developmental stage was confirmed by visual inspection of eye pigmentation and length of limbs according to the Hamburger-Hamilton staging system^3^. The border between the air sac and the amniotic space was marked out, and a hole made into the shell over the air sac by thin forceps roughly 1 mm away from the marked-out border. One tip from a pair of thin forceps was inserted into the hole, tangentially to the egg, and the inside of the egg was gently scraped, perforating the sac membrane in order shift the air bubble to above the embryo. Upon successful shifting of the air bubble, as confirmed by the embryo no longer being visible when candling, a piece of surgical tape (1530-1, VWR, Radnor, PA, USA) was placed over the presumed position of the embryo. The tape and underlying egg shell were pierced and a hatch was cut out to expose the embryo. The vitelline and amniotic membranes were gently peeled open with bent forceps to expose the eye of the embryo. St22/E3.5 chicken eyes were electroporated with plasmids (1-2µg/µl per vector) in 1x PBS with Ca²⁺ and Mg²⁺ (18912-014, Gibco, Waltham, MA, USA; henceforth referred to as “PBS+/+”). Fast Green (F7252, Sigma-Aldrich, St. Louis, MO, USA,) was added to injection solution for visualization of the injection. 0.2 μl solution was manually injected into the subretinal space of the central retina by the use of a capillary and mouth pipetting. The capillary was inserted dorsally, next to the temporal long posterior ciliary artery and on the border of the eye and the prospective brain. A negative electrode was placed behind the central region of the eye, by the injected plasmid solution, and a positive electrode was placed between the eye and the prospective beak. Five 50 ms pulses of 15 V with 1 sec. intervals were applied using an ECM 830 Square pulse electroporator (BTX Harvard Apparatus, Holliston, MA, USA). Electrodes were gently removed and 100 µl of Ringer’s solution was gently applied to the electroporated eye via pipetting. The hatch was closed and sealed with another piece of surgical tape and the egg incubated for continued development.

For animals intended for hatching, the same protocol was followed with the following changes: the position of the air sac was not manipulated and surgical tape was not used. Instead, a small square encompassing the embryo was gently filed out manually. The shell was gently lifted out and the membranes peeled back. Following electroporation and addition of Ringer’s solution, the membranes were returned and the shell was placed back in its original position. The shell was glued back on and the egg placed into a humidified ECO 3 DU incubator (Maino Industries, Oltrona di San Mamette, Italy) at 37°C, which tilts the eggs rather than rotating them. One day prior to hatching, eggs were transferred to flat-surfaced cages to provide suitable surface for hatching, also at 37°C in a humidified incubator.

Retinoids were electroporated in 1 mm cuvettes (732-0020, VWR) with 1.67 µg/µl DNA of each vector in PBS+/+ (14080048, Gibco). Retinoids of similar age, size, and morphology were selected at differentiation day 39-41. Three to six retinoids were electroporated using the ECM 830 Square pulse electroporator with the same program used *in ovo*. Retinoids were further cultured in ULA plates (3471, Corning, New York, NY, USA) in fresh differentiation medium.

## Preparation of samples for cell cycle analysis using Guava easyCyte Flow cytometer

St34/E8 and st40/E14 chicken retinas and established MYCN cells were prepared for cell cycle analysis using an easyCyte 8 Flow cytometer, as follows. The central part of the retina was dissected and incubated in 1x Trypsin-EDTA (15400054, Gibco) at 37°C for 5 minutes. An equal volume of FBS (16000044, Gibco) was added to terminate the reaction. Cultured retinal cells were centrifuged at 300 rcf for 5 minutes, the medium aspirated, and the cells incubated in 1x PBS without Ca²⁺ and Mg²⁺ (14190094, Gibco; henceforth referred to as “PBS-/-”) Single-cell suspensions were obtained in both cases by gentle trituration, one wash in PBS-/-, and filtration through a 20 µm cell strainer (43-10020-60, pluriSelect, Leipzig, Germany). Cell densities were counted in a Bürker chamber and 1 × 10⁶ cells were used per sample. The samples were washed once with PBS-/- and resuspended in 500 µl PBS-/-. To fix GFP, 500 µl of 2% PFA was added to each sample, followed by incubation at 4°C for 1 hour. The samples were centrifuged at 300 rcf for 5 minutes, the supernatant aspirated, and the samples washed once in PBS-/-. One millilitre of ice cold 70% ethanol was added dropwise under agitation and the samples were incubated at 4°C overnight. The ethanol was aspirated following centrifugation at 1000 rcf for 5 minutes. The samples were resuspended in propidium iodide (PI) working solution (0.1% TritonX-100, 10 µg/ml PI; P4864, Sigma-Aldrich, St. Louis, MO, USA), and 100 µg/ml DNase-free RNase A (11119915001, Sigma-Aldrich) in PBS-/- and incubated at room temperature for 30 minutes before analysed with the easyCyte 8. Aggregates or doublets were excluded from analysis. Fixed, non-electroporated retinal cells were used as a negative control to set the threshold for a positive signal. Propidium iodide (PI; P4864, Sigma-Aldrich) or GFP positive cells were gated after compensation with single-color controls, which were GFP positive cells in culture and non-electroporated retinal cells with PI staining.

## Quantitative reverse transcriptase PCR analysis

The central region of the retina was taken in retina older than st29/E6 to avoid bias imposed by the centro-peripheral aspects of retinal development. For all treatments/stages, a minimum of at least four animals were analysed. The samples were DNase-treated (M6101, Promega, Madison, WI, USA) and cDNA was synthesized with the High-capacity RNA-to-cDNA kit (4387406, Thermo Fisher Scientific, Waltham, MA, USA). Tests were run in duplicates using IQ™ SYBR® green Supermix (1708882, Bio-Rad Laboratories AB, Hercules, CA, USA) and the C_t_ values were normalized to β-actin and TBP (TATA box-binding protein). Control reactions containing Supermix and primers but no cDNA were run in parallel. PCR program consisted of initial denaturation step at 95°C for 3 minutes, followed by 39 cycles of denaturation at 95°C for 15 seconds, and annealing and extension at 60°C for 30 seconds. Melt curve analysis was performed to confirm the presence of a single product. Negative controls without template were included in every plate for each primer mix to screen for unspecific amplification. The primers (Supplementary Table S2) were designed with Primer Express v2.0 (Applied Biosystems, Darmstadt, Germany).

## PiggyBac vector integration analysis

Quantitative PCR analysis with primers against the 5’-ITR of the piggyBac transposon and two single-copy reference genes (Supplementary Table S2) were used to analyse the number of integrations resulting from the *in ovo* E3.5 electroporation. Genomic DNA was extracted and fragmented by repeated freeze-thaw cycles. Fragmented gDNA was serially diluted to a 0.05 ng/µl stock for each sample and then used to produce six dilutions in a 2-fold dilution series. Each sample and its respective dilutions were subjected to qPCR analysis with primers against the 5’-ITR of the piggyBac transposon as well as proopiomelanocortin (POMC) and pro-melanin concentrating hormone (PMCH). Results from POMC and PMCH provide a relative baseline as one copy per haploid genome from which the number of integrations per haploid genome can be calculated. C_t_ values for each dilution and primer pair were plotted to verify linearity in amplification (See Supplementary Fig S3A). ΔC_t_ was obtained by subtracting the average C_t_ of POMC and PMCH (the relative baseline) from the C_t_ for the 5’-ITR for each dilution. The number of integrations per haploid genome were calculated by 2^-ΔCt^ and an average of all 2^-ΔCt^-values across the dilution series was used as an estimate of the number of transgene integrations per haploid genome (See Supplementary Fig S3B).

## Human embryonic stem cells and retinoid differentiation protocol

Human embryonic stem cells (hESCs) cell line HS980^4, 5^ (Ethical permit number EPN-20117745-31/3) were maintained on human recombinant laminin LN521^6^ (Biolamina, Sundbyberg, Sweden) in 24-well plates (83.3922.005, Sarstedt, Nümbrecht, Germany) NutriStem hESC XF medium (05-100-1A, Biological industries, Beit Haemek, Israel) at 37°C, 5% CO₂. The cells were passaged every 5-7 days, or when they reached 90-100% confluence using TrypLE Select (12563011, Gibco). To confirm genomic integrity, the karyotype was analysed at Ambarlab (Hospitalet de Llobregat, Barcelona, Spain) after colcemid treatment and fixation with Carnoy’s solution (3:1 methanol:glacial acetic acid). The karyotype was normal. Genetic analysis for regional amplifications was also performed with hESC Genetic Analysis Kit (#07550, StemCell Technologies, Vancouver, Canada) and found to be normal.

Once the stem cells reached 100% confluence (day 0; D0), differentiation was initiated by washing and incubation in PBS-/- at 37°C for 5 minutes, to loosen cell-laminin contacts. Small cell clumps were generated by replacing the PBS-/- with NutriStem hESC XF (05-100-1A, Biological industries) supplemented with 10 µM Blebbistatin (B0560, Sigma-Aldrich) and gently scraping the dish in a grid-like pattern with a 1 ml pipette tip until all cells were in suspension. The cell clumps were transferred to 6-well ULA plates (3471, Corning) with NutriStem and 10 µM Blebbistatin to initiate formation of embryoid bodies (EBs). The EBs were gradually transitioned into neural induction medium (NIM; 11320-074, DMEM/F12 1:1, 17502001, 1% N2 supplement, 11140035, 1% MEM-NEAA, 15140122, 1% pen:strep, Gibco, and H3393, 2 µg/ml heparin, Sigma-Aldrich) by replacing 50% of the medium on D1-3 and D5. On D1, the fresh medium consisted of a 1:1 ratio of NutriStem and NIM, and on D2, D3, and D5 of 100% NIM. On D7, the cell aggregates were seeded onto growth factor-reduced Matrigel (354230, Corning) in NIM. From D9 onwards, the medium was replaced daily. On D16, NIM was substituted for differentiation medium (41966-029, DMEM and 21765-029, F12; 3:1, 12587001 2% B27 supplement without vitamin A, 15240062, 1% MEM-NEAA, and 15240062, 1% antibiotic-antimycotic, Gibco). Neural retinal domains were manually dissected with 24G needles under an inverted microscope on D20-28. The resulting retinoids were cultured in suspension, 5-10 retinoids/well in 6-well ULA plates. On D42 and onwards, the differentiation medium was supplemented with 10% FBS, 1% GlutaMAX (35050061, Gibco), and 12.5 µg/ml Taurine (T0625, Sigma-Aldrich). The protocol followed the one published by Zhong et al with minor changes^7^.

## Immuno- and histochemistry

Dissected chicken eyes and human retinoids were fixed, embedded, and sectioned. Retinoids and retinal tissue were washed in 1x PBS+/+ for 5 minutes, fixed in 4% PFA for 15 minutes, washed in 1x PBS+/+ for 10 minutes, and cryoprotected in 30% sucrose overnight (or for 3-4 hours for retinoids). All steps were performed at 4°C. The tissues and retinoids were embedded in OCT (Neg-50, 6502, Thermo Fisher Scientific), frozen on dry ice, and 10 µm sections were collected on Superfrost Plus slides (J1800AMNZ, Menzel-Gläser, Germany).

Sections were rehydrated in 1x PBS+/+ for 10 minutes prior to incubation in blocking solution (1% FBS, 0.1% Triton X-100, and 0.02% thimerosal in 1x PBS+/+ for 30 minutes at room temperature. The sections were incubated in primary antibody solution at 4°C overnight in a humidified chamber, washed 3 × 5 minutes in 1x PBS+/+ at room temperature, and incubated in secondary antibody solution for ≥2 hours at room temperature. Dilutions were in blocking solution. For antibodies and dilutions see Supplementary Table S3. Slides were mounted and nuclei visualized using ProLong Gold Antifade Mountant with DAPI (P36935, Invitrogen, Waltham, MA, USA). Immunochemistry included negative controls (without primary and with secondary antibody) and positive controls (Sections with well-known IR patterns) (data not shown).

TUNEL Plus staining (C10619, Invitrogen) was performed according to the manufacturer’s instructions.

For haematoxylin and eosin staining, sections were allowed to reach room temperature before fixation in 4% PFA for 15 minutes. Sections were incubated for three minutes each in 70% and then 99.5% ethanol before incubation in haematoxylin and eosin solution (diluted 1:4 in tap water) for 15 minutes. Sections were washed in tap water for 5 minutes, differentiated in 70% ethanol with 0.1% HCl, left to dry, and mounted in ProLong Gold Antifade Mountant without DAPI (P36934, Invitrogen).

## Microscopy, image analysis, cell counting and statistical analysis

Images of enucleated chicken eyes were captured using a Leica M165FC fluorescence stereomicroscope equipped with a Leica DFC495 camera (Leica, Wetzlar, Germany). Fluorescence images of sectioned tissues and cells were captured using a Zeiss Axioplan 2 microscope equipped with an AxioCam HRc camera or a Zeiss Imager Z2 microscope equipped with an Axiocam 512 monochromatic camera (Carl Zeiss Microscopy GmbH, Jena, Germany).

After explorative examinations, experiments that aim to quantify eg. developmental changes in cell number, are designed with six animals so that at four biological replicates can be analyzed even if animals or analyses were unsuccessful.

Animals were included in the study if: 1. they survived to the intended developmental stage; 2. if they contained GFP+ cells after electroporation to be deemed a successful electroporation and usable for IHC or RNA extraction (based on size of GFP+ area in the retina). 3. If the IHC analysis proved to have worked as judged by inspection of non-electroporated regions in the retina. Manual counting of cells was performed in Fiji based on ImageJ^8^ and the experimental designs included counting of at least four histological sections from each of four independently dissected retina samples (biological replicates, n=4, see figure legends for specifics). The number of cells is indicated in the figure legends, typically total number GFP+ cells that were counted. Sample counting was not performed blinded to the group. The data were analysed with one-way ANOVA followed by Tukey’s multiple comparison post-hoc test or Student’s *t* test using GraphPad Prism (GraphPad software Inc.) and statistical significance was set to *p* < 0.05. Normal distribution of samples was assumed and tested. Figures were assembled in Adobe Photoshop and images were adjusted for contrast to more clearly show the immunohistology (Adobe Systems Incorporated, San Jose, CA, USA). Micrographs were selected to show a representative image of four analyzed samples and in many samples an over-view image together with a high magnification micrograph are shown.

Live retinoids were observed and photographed using a Zeiss Axiovert S100 inverted microscope equipped with an AxioCam HRc camera. For this, the retinoids were briefly transferred to plates containing PBS-/- before returning to wells with differentiation medium and further cultured at 37°C, 5% CO₂.

Analysis of co-localization of immunoreactivity (IR) for GFP^nuc^ (MYCN) and pan-Brn3, Lim1, or Otx2 were performed on fluorescence micrograph images of sections of retinoids (Fig. 5E) using the colocalization feature in CellProfiler (v4.2)^8, 9, 10^. Regions were first identified based on both IR of GFP and the second cell marker of interest. A region of interest (ROI) was then defined based on the GFP IR and the pixel correlation between fluorescence channels Alexa 488 (GFP) and Alexa 568 (pan-Brn3, Lim1, or Otx2) in the ROI was calculated. Colocalization was expressed using Pearson’s correlation coefficient (PCC). Three immunochemical sections of four retinoids from each time-point were analysed. For hypothesis testing, one of the analysed channel-images was rotated 90° and measured again. Images with PCC of 0.5 decreased after rotation, typically to <0.2, and the hypothesis was verified. The PCC ranges from 0 to 1, where 0 is no correlation and 1 is high correlation. Values varied typically between 0.1 to 0.8 and <0.2 was considered as low correlation.

## RNA sequencing

## Total RNA from three samples each of MYCN cells and st40/E14 retina was extracted with Qiagen RNeasy Micro Kit (#74004, Qiagen, Hilden Germany) following manufacturer’s instructions. The library establishment and RNA sequencing were performed by SNP&SEQ Platform of National Genomics Infrastructure in Sweden. The library was prepared using the TruSeq Stranded mRNA Library Preparation Kit with polyA selection (Illumina Inc., San Diego, CA, USA). Sequencing was performed on SP Flow Cell using the NovaSeq 6000 system and v1.5 sequencing reagents (Illumina Inc.).

## Analysis of RNA sequencing data

For detection and removal of rRNA contamination bbduk from BBMap (version 38.61)^11^ was used. Quality check and alignment of the data was done by using the nf-core/rnaseq pipeline (version 3.4)^12^ by adjusting the alignment parameter (--alignEndsProtrude 100 ConcordantPair) to perform QC and alignment of the data. The first 10 bp of the reads were hard-clipped due to biased base composition. MultiQC ^13^and FastQC^14^ results reported by nf-core pipeline were used for quality control. To extract fragment counts, featureCounts (version 2.0.0)^15^ was used and a minimum mapping quality of 20 was imposed and required both pairs to be properly aligned on the same chromosome. Pairwise comparison by using edgeR^16^ with pairwise comparison (exactTest) based on E14 retina and MYCN cells, was performed. Significant differentially expressed genes (DEG) were selected based on two criteria: 1) corrected p-value for multiple testing (FDR < 0.05, Benjamini-Hochberg method) and 2) log2 fold-change > 1. GO gene-set enrichment analysis was performed: DEGs were analysed by Clusterprofiler (version 4.0.6)^17^ and gseGO^17^. Terms with at least 10 genes were used for downstream analysis and visualisation. All analyses were performed using R Statistical Software (v4.1.1, R Core Team 2021)^18^. All codes are available upon request. The computations were performed on resources provided by SNIC through Uppsala Multidisciplinary Center for Advanced Computational Science (UPPMAX) under Project snic2021-22-505 (Computation) & snic2021-23-425 (Storage). Support was given from Science for Life Laboratory, the National Genomics Infrastructure, NGI, and Uppmax by providing computational infrastructure for massive parallel sequencing.

**References**

1. Alexopoulou AN, Couchman JR, Whiteford JR. The CMV early enhancer/chicken beta actin (CAG) promoter can be used to drive transgene expression during the differentiation of murine embryonic stem cells into vascular progenitors. *BMC Cell Biol* 2008, **9:** 2.

2. Blixt MK, Hallböök F. A regulatory sequence from the *retinoid X receptor γ gene* directs expression to horizontal cells and photoreceptors in the embryonic chicken retina. *Mol Vis* 2016, **22:** 1405-1420.

3. Hamburger V, Hamilton H. A series of normal stages in the development of the chicken embryo. *J Morphol* 1951, **88**(3)**:** 49-92.

4. Rodin S, Antonsson L, Hovatta O, Tryggvason K. Monolayer culturing and cloning of human pluripotent stem cells on laminin-521-based matrices under xeno-free and chemically defined conditions. *Nat Protoc* 2014, **9**(10)**:** 2354-2368.

5. Rodin S, Antonsson L, Niaudet C, Simonson OE, Salmela E, Hansson EM*, et al.* Clonal culturing of human embryonic stem cells on laminin-521/E-cadherin matrix in defined and xeno-free environment. *Nature communications* 2014, **5:** 3195.

6. Plaza Reyes A, Petrus-Reurer S, Antonsson L, Stenfelt S, Bartuma H, Panula S*, et al.* Xeno-Free and Defined Human Embryonic Stem Cell-Derived Retinal Pigment Epithelial Cells Functionally Integrate in a Large-Eyed Preclinical Model. *Stem Cell Reports* 2016, **6**(1)**:** 9-17.

7. Zhong X, Gutierrez C, Xue T, Hampton C, Vergara MN, Cao LH*, et al.* Generation of three-dimensional retinal tissue with functional photoreceptors from human iPSCs. *Nature communications* 2014, **5:** 4047.

8. Schindelin J, Arganda-Carreras I, Frise E, Kaynig V, Longair M, Pietzsch T*, et al.* Fiji: an open-source platform for biological-image analysis. *Nat Methods* 2012, **9**(7)**:** 676-682.

9. Jones TR, Kang IH, Wheeler DB, Lindquist RA, Papallo A, Sabatini DM*, et al.* CellProfiler Analyst: data exploration and analysis software for complex image-based screens. *BMC Bioinformatics* 2008, **9:** 482.

10. Lamprecht MR, Sabatini DM, Carpenter AE. CellProfiler: free, versatile software for automated biological image analysis. *Biotechniques* 2007, **42**(1)**:** 71-75.

11. Bushnell BUS. BBMap: A Fast, Accurate, Splice-Aware Aligner. 2014, [**https://www.osti.gov/servlets/purl/1241166**](https://www.osti.gov/servlets/purl/1241166).

12. Patell H, Ewels P, Peltzer A, Hammarén R, Botvinnik O, Sturm G. nf-core/rnaseq: nf-core/rnaseq v3.4 - Platinum Platypus. 2021, **doi:10.5281/zenodo.5550247**.

13. Ewels P, Magnusson M, Lundin S, Kaller M. MultiQC: summarize analysis results for multiple tools and samples in a single report. *Bioinformatics* 2016, **32**(19)**:** 3047-3048.

14. Andrews S, Krueger F, Segonds-Pichon A, Biggins L, Krueger C, Montgomery J. FastQC: A Quality Control tool for High Throughput Sequence Data. 2019, [**http://www.bioinformatics.babraham.ac.uk/projects/fastqc/**](http://www.bioinformatics.babraham.ac.uk/projects/fastqc/).

15. Liao Y, Smyth GK, Shi W. featureCounts: An efficient general purpose program for assigning sequence reads to genomic features. *Bioinformatics* 2013, **30**(7)**:** 923-930.

16. Robinson MD, McCarthy DJ, Smyth GK. edgeR: a Bioconductor package for differential expression analysis of digital gene expression data. *Bioinformatics* 2010, **26**(1)**:** 139-140.

17. Yu G, Wang LG, Han Y, He QY. clusterProfiler: an R package for comparing biological themes among gene clusters. *OMICS* 2012, **16**(5)**:** 284-287.

18. R Core Team. R: A Language and Environment for Statistical Computing, Vienna, Austria. 2021, [**https://www.R-project.org/**](https://www.R-project.org/).
