# Supplementary material for: MYCN induces cell-specific tumorigenic growth in RB1-proficient human retinal organoid and chicken retina models of retinoblastoma
Source: Oncogenesis. 2022 Jun 21;11(1):34. doi: 10.1038/s41389-022-00409-3 (PMC9213451; doi:10.1038/s41389-022-00409-3)

Supplementary figure S1

*MYCN* induces tumorigenic growth in *RB1*-proficient human retinal organoid- and chicken retina models of retinoblastoma.

Maria K E Blixt, Minas Hellsand, Dardan Konjusha, Hanzhao Zhang, Sonya Stenfelt, Mikael Åkesson, Nima Rafati, Tatsiana Tararuk, Gustav Stålhammar, Charlotta All-Eriksson, Henrik Ring, and Finn Hallböök.

***Complementary micrographs of E14 chick retina after electroporation with MYCN, c-Myc or control vectors.***

Micrographs of E14 retina that was electroporated at E3.5 with the indicated vectors and stained for visinin (Vis), Lim1/2 (Lim1), Ap2α and Brn3a. Expression vectors were a) CAG-MYCN, b) CAG-MYCN^T58A^ and c) CAG-c-Myc^T58A^. CAG is the chicken actin gene promotor and drives strong, ubiquitous expression in all cell types in the retina. d) MYCN^T58A^ expression driven by CAG that was specifically directed to cells by a 208 bp RXRγ-regulatory gene element and e) a control with GFP-only expression also directed by the RXRγ-regulatory gene element. The RXRγ-regulatory gene element drive expression to cone photoreceptor/horizontal progenitors (Blixt and Hallböök 2016) and we used the RXRγ-element to drive Cre expression to these progenitors. Cre excises a “floxed” transcriptional Stop signal (loxP-Stop-loxP), which then released the expression from the CAG promoter. f) Immunohistochemistry for phospho-histone H3 (PH3). Abbreviations: gc; ganglion cells, gcl; ganglion cell layer, hc; horizontal cells, (hc -Lim1 strong/weak; strong defines differentiated hc in the hc layer, weak defines progenitors), inl; inner nuclear layer, onl; outer nuclear layer, os; outer photoreceptor segments

Blixt, M. K. E. & F. Hallböök (2016) A Regulatory Element from the Retinoid X Receptor γ Gene Directs Expression to Horizontal Cells and Photoreceptors in the Embryonic Chicken Retina. *Mol Vis,* 22**,** 1405-1420.


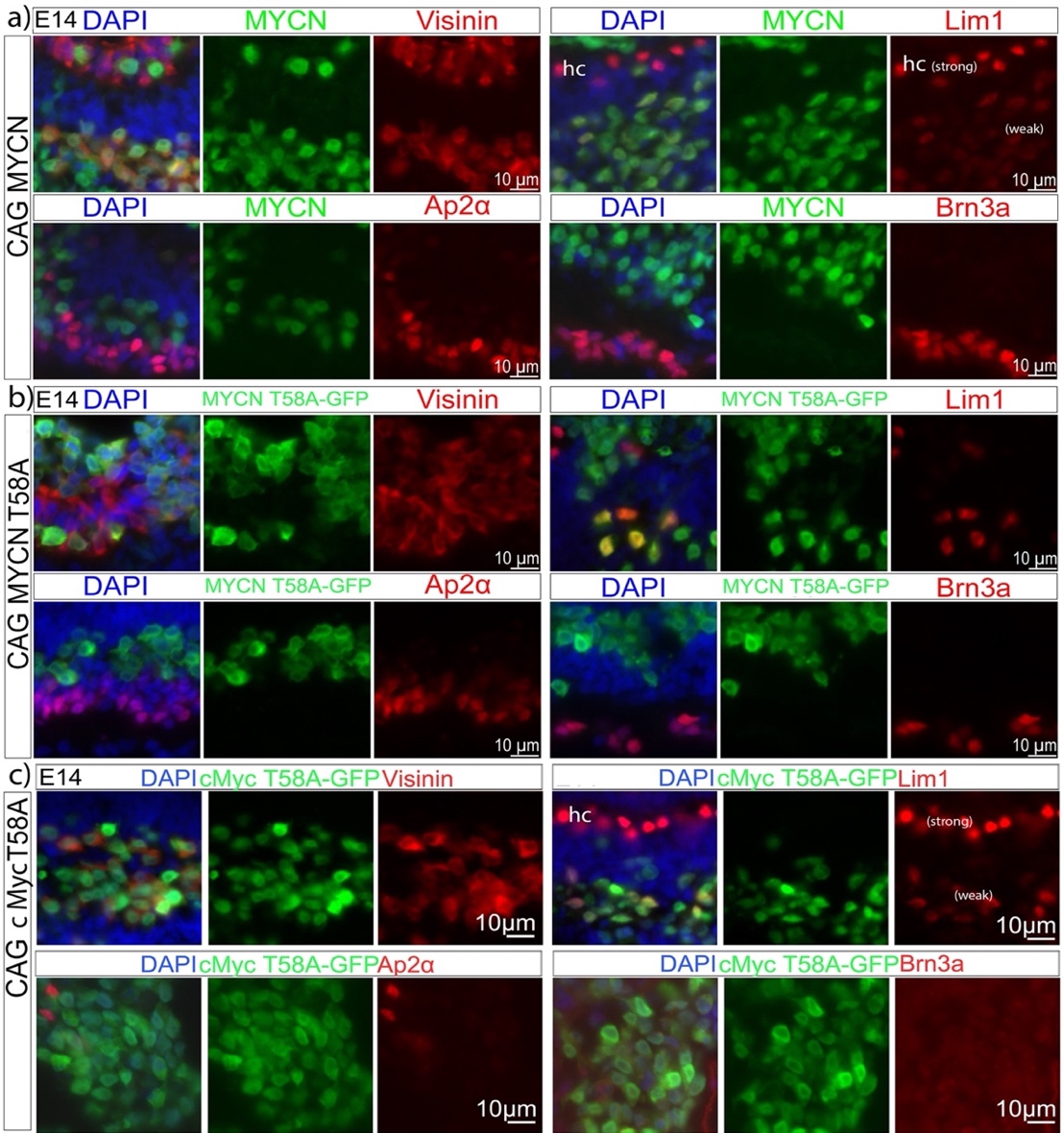


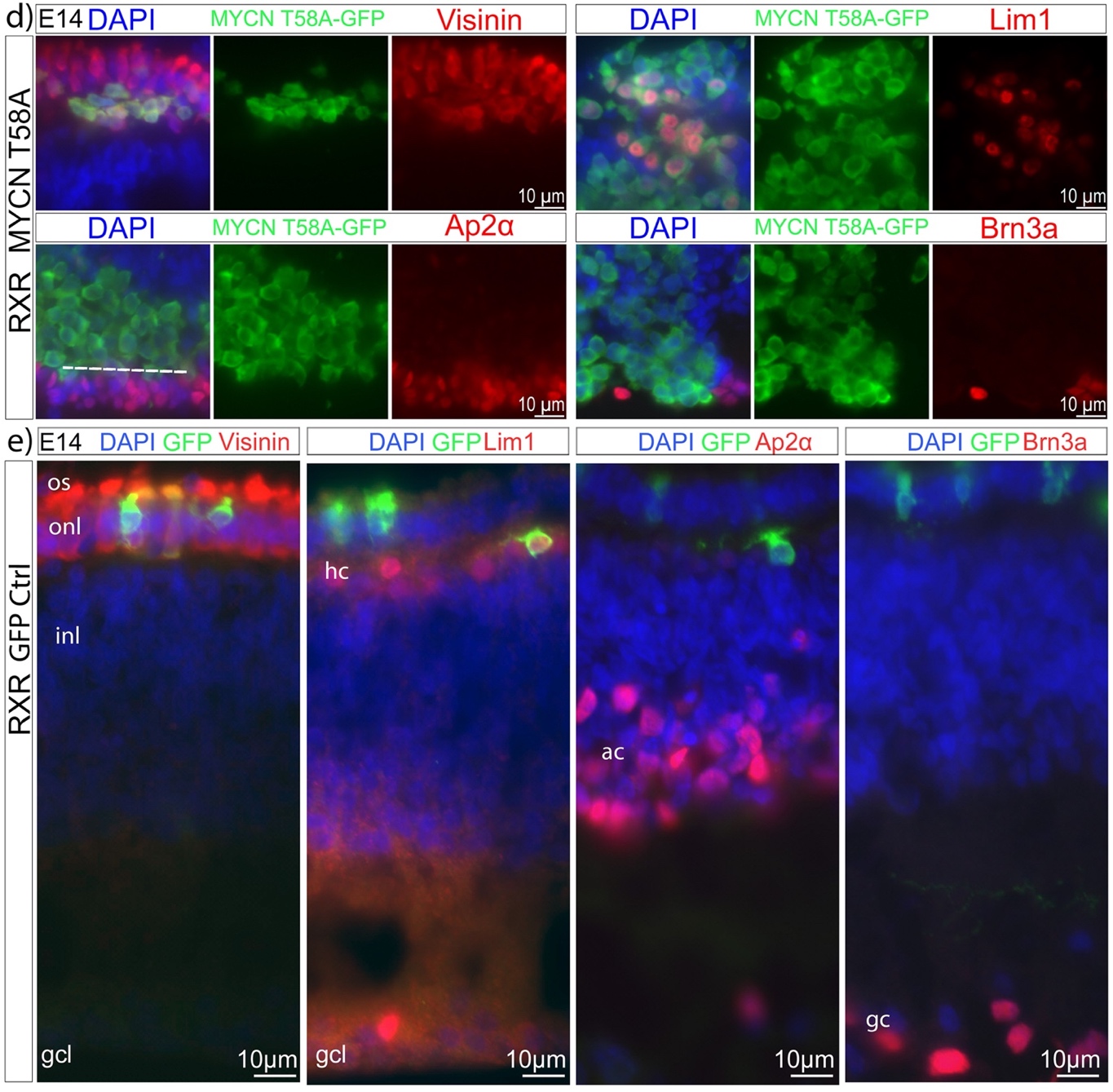


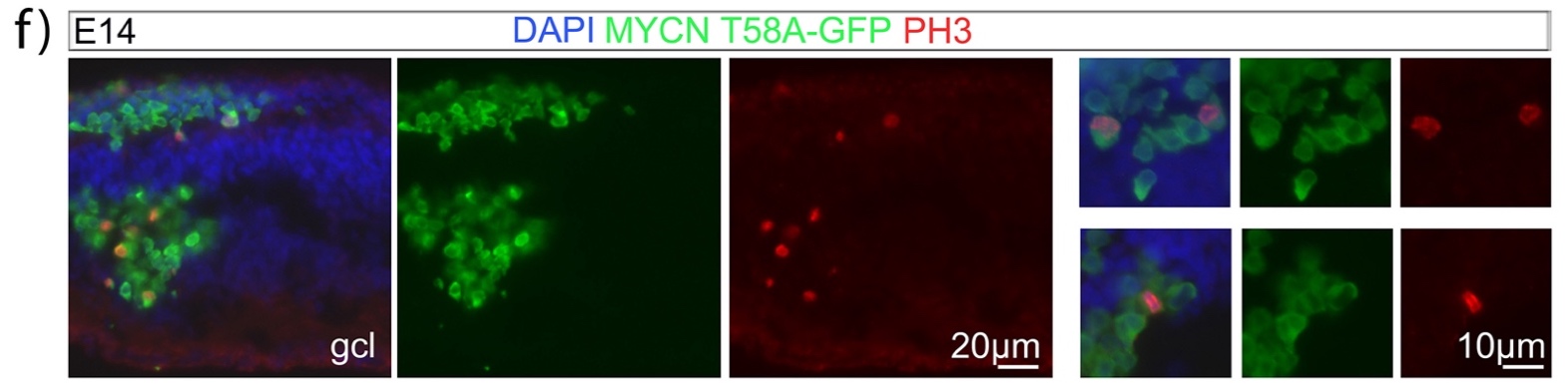

Supplement: Supplementary file 2 — Supplementary figure S1 [file 41389_2022_409_MOESM2_ESM.docx]
