# Supplementary material for: MYCN induces cell-specific tumorigenic growth in RB1-proficient human retinal organoid and chicken retina models of retinoblastoma
Source: Oncogenesis. 2022 Jun 21;11(1):34. doi: 10.1038/s41389-022-00409-3 (PMC9213451; doi:10.1038/s41389-022-00409-3)
Supplement: Supplementary file 3 — Supplementary figure S2A [file 41389_2022_409_MOESM3_ESM.docx]

Supplementary figure S2A

*MYCN* induces tumorigenic growth in *RB1*-proficient human retinal organoid- and chicken retina models of retinoblastoma.

Maria K E Blixt, Minas Hellsand, Dardan Konjusha, Hanzhao Zhang, Sonya Stenfelt, Mikael Åkesson, Nima Rafati, Tatsiana Tararuk, Gustav Stålhammar, Charlotta All-Eriksson, Henrik Ring, and Finn Hallböök.

***Fig. S2A. Stage-specific effects of MYCN^T58A^ or c-MYC in chicken retina***

Bar graphs with fractions of GFP, Lim1 and GFP, visinin (Vis) double-positive cells in retinas electroporated with *MYCN^T58A^* and control (ctrl) or c-*MYC^T58A^* and control group retinas. The retinas were electroporated *in ovo* at st22 (E3.5), st25 (E4.5), and st28 (E6) with cell counts of double+ cells after 48 hours. Anova, Mean +/- SD, n=4, * *p* ˂ 0.05
