# Supplementary material for: MYCN induces cell-specific tumorigenic growth in RB1-proficient human retinal organoid and chicken retina models of retinoblastoma
Source: Oncogenesis. 2022 Jun 21;11(1):34. doi: 10.1038/s41389-022-00409-3 (PMC9213451; doi:10.1038/s41389-022-00409-3)

Supplementary figure S2B

*MYCN* induces tumorigenic growth in *RB1*-proficient human retinal organoid- and chicken retina models of retinoblastoma.

Maria K E Blixt, Minas Hellsand, Dardan Konjusha, Hanzhao Zhang, Sonya Stenfelt, Mikael Åkesson, Nima Rafati, Tatsiana Tararuk, Gustav Stålhammar, Charlotta All-Eriksson, Henrik Ring, and Finn Hallböök.

***Fig. S2B. TUNEL staining of E10 chick retina with MYCN expression.***

Fluorescence micrographs of E10 retina that was electroporated at st22 (E3.5) with the CAG driven MYCN expression vector and stained for Ap2α in combination with TUNEL staining. Control is a GFP-negative region in the same eye. Arrowheads indicate triple-positive cells. a) and b) panels are magnifications of regions in the dashed line boxes in the top MYCN-GFP panel. GFP in green, AP2α in red, TUNEL in white and DAPI in the blue channel. Abbreviations: gcl; ganglion cell layer, inl; inner nuclear layer, onl; outer nuclear layer.


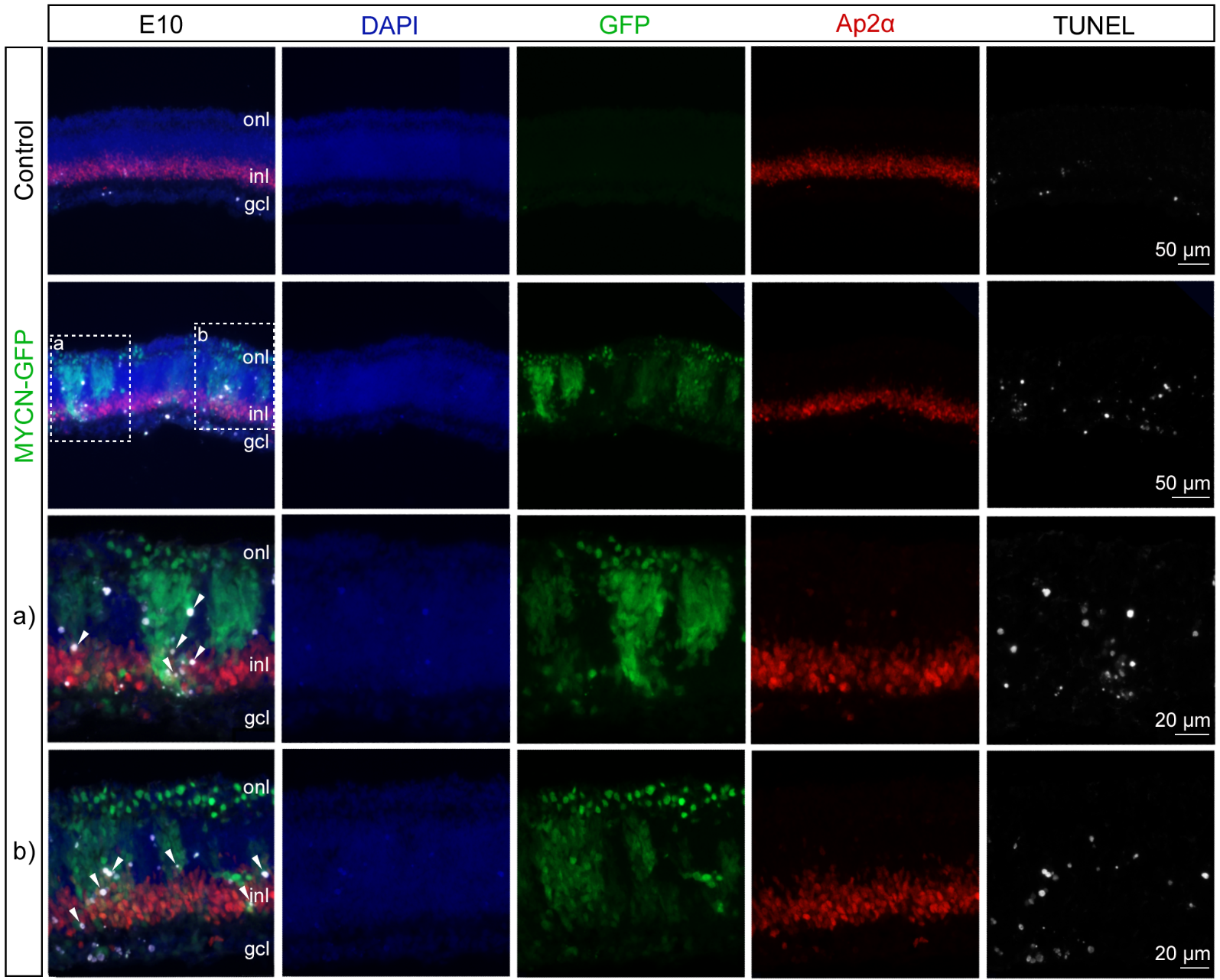

Supplement: Supplementary file 4 — Supplementary figure S2B [file 41389_2022_409_MOESM4_ESM.docx]
