# Supplementary material for: MYCN induces cell-specific tumorigenic growth in RB1-proficient human retinal organoid and chicken retina models of retinoblastoma
Source: Oncogenesis. 2022 Jun 21;11(1):34. doi: 10.1038/s41389-022-00409-3 (PMC9213451; doi:10.1038/s41389-022-00409-3)
Supplement: Supplementary file 5 — Supplementary figure S3A [file 41389_2022_409_MOESM5_ESM.docx]

Supplementary figure S3A

*MYCN* induces tumorigenic growth in *RB1*-proficient human retinal organoid- and chicken retina models of retinoblastoma.

Maria K E Blixt, Minas Hellsand, Dardan Konjusha, Hanzhao Zhang, Sonya Stenfelt, Mikael Åkesson, Nima Rafati, Tatsiana Tararuk, Gustav Stålhammar, Charlotta All-Eriksson, Henrik Ring, and Finn Hallböök.

***Fig. S3A. GFP, Ki67 and E2F1 mRNA levels in MYCN cells and E14 retina***

qRT-PCR analysis with primers against GFP, Ki67 and E2F1. qRT-PCR was performed to validate findings from RNA sequencing performed on MYCN amplified cells derived from electroporated retina.

***
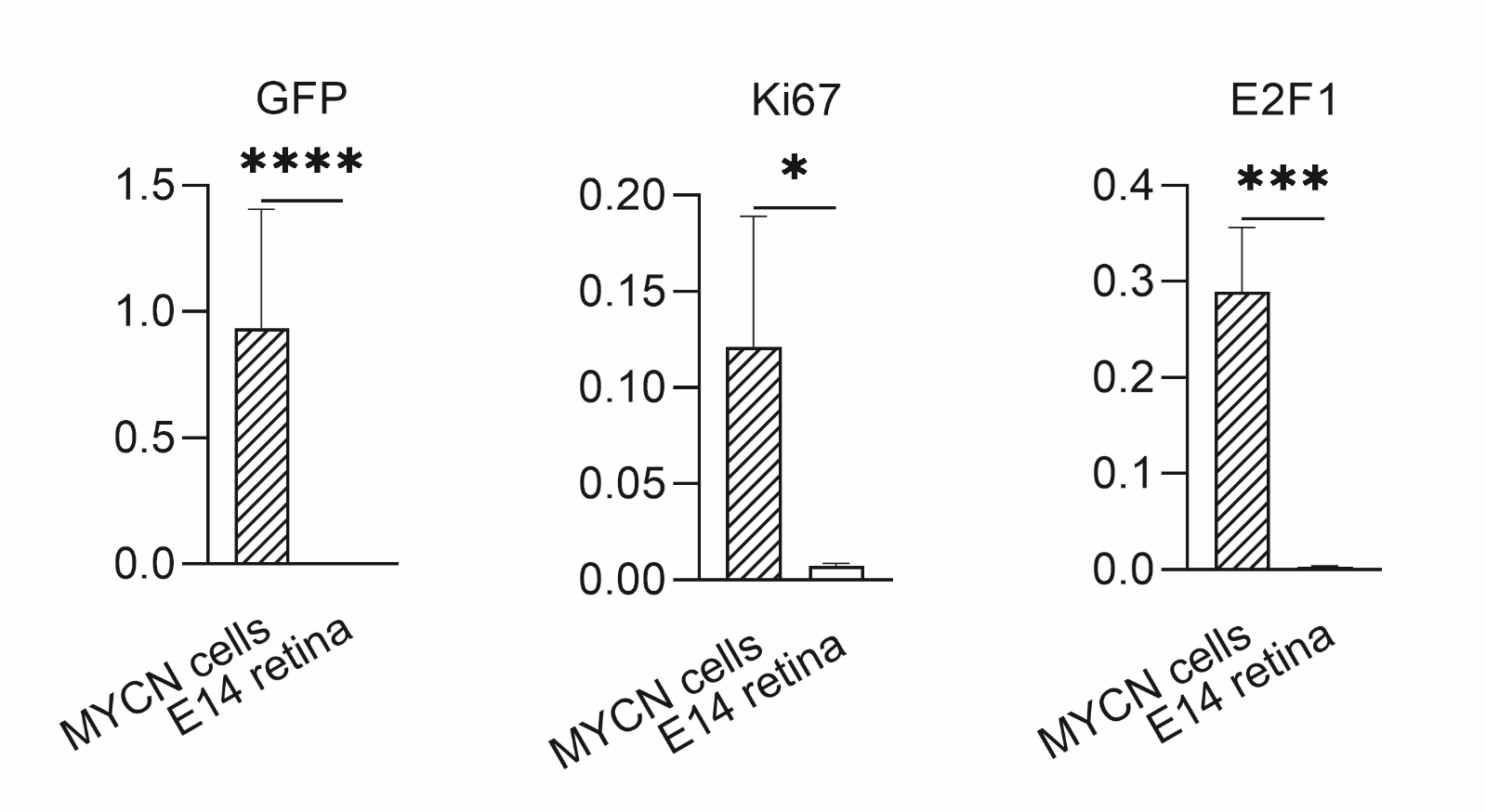
***
