# Supplementary material for: MYCN induces cell-specific tumorigenic growth in RB1-proficient human retinal organoid and chicken retina models of retinoblastoma
Source: Oncogenesis. 2022 Jun 21;11(1):34. doi: 10.1038/s41389-022-00409-3 (PMC9213451; doi:10.1038/s41389-022-00409-3)
Supplement: Supplementary file 6 — Supplementary figure S3B [file 41389_2022_409_MOESM6_ESM.docx]

Supplementary figure S3B

*MYCN* induces tumorigenic growth in *RB1*-proficient human retinal organoid- and chicken retina models of retinoblastoma.

Maria K E Blixt, Minas Hellsand, Dardan Konjusha, Hanzhao Zhang, Sonya Stenfelt, Mikael Åkesson, Nima Rafati, Tatsiana Tararuk, Gustav Stålhammar, Charlotta All-Eriksson, Henrik Ring, and Finn Hallböök.

***Fig. S3B. Determination of piggyBac integrations per haploid genome***

Quantitative PCR analysis with primers against the 5’ ITR of the piggyBac transposon and single-copy reference genes (*POMC* and *PMCH*) was used to analyze the number of integrations as a result of *in ovo* E3.5 electroporation.

a) C_t_ values for each dilution and primer pair plotted to verify linearity in amplification and calculating integration number in genomic DNA from established cells and electroporated retina. ΔC_t_ was obtained by subtracting the average C_t_ of the relative baseline from the C_t_ for the 5’-ITR for each dilution. “DMC-1 -7” are established primary cell lines and “MYCN el. E14 retina” are gDNA prepared from dissected electroporated regions of E14 retina. Note that in the dissected retina only approximately 1% of the cells carries integrations (see supplm. fig S3C).

b) The number of integrations per haploid genome resulting from *in ovo* E3.5 (st22) electroporation as calculated by averaging all 2-ΔC_t_ values across the dilution series. The relative baseline was established from the single-copy genes POMC and PMCH.


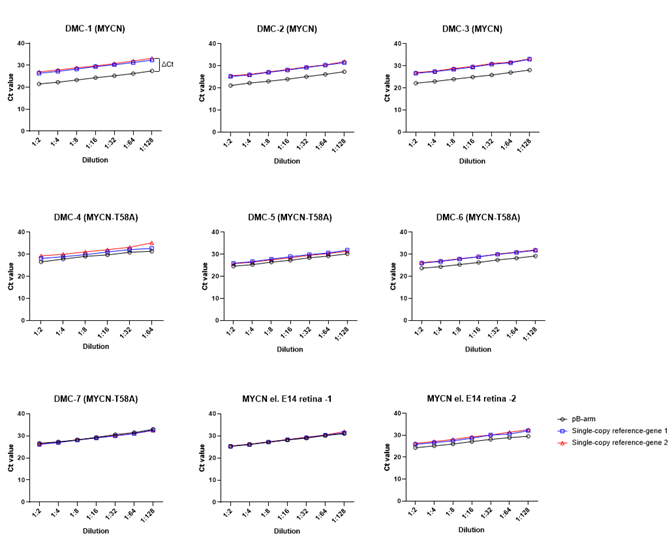


a)


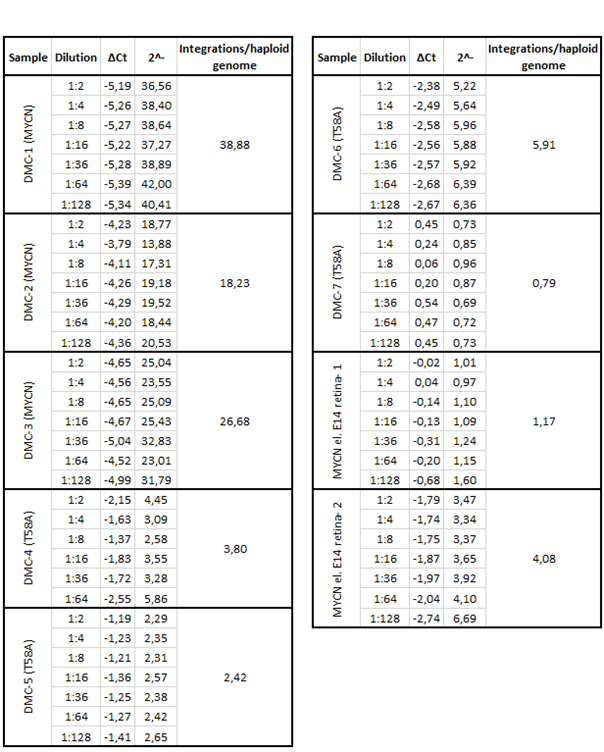


b)
