# Supplementary material for: MYCN induces cell-specific tumorigenic growth in RB1-proficient human retinal organoid and chicken retina models of retinoblastoma
Source: Oncogenesis. 2022 Jun 21;11(1):34. doi: 10.1038/s41389-022-00409-3 (PMC9213451; doi:10.1038/s41389-022-00409-3)

Supplementary figure S3C

*MYCN* induces tumorigenic growth in *RB1*-proficient human retinal organoid- and chicken retina models of retinoblastoma.

Maria K E Blixt, Minas Hellsand, Dardan Konjusha, Hanzhao Zhang, Sonya Stenfelt, Mikael Åkesson, Nima Rafati, Tatsiana Tararuk, Gustav Stålhammar, Charlotta All-Eriksson, Henrik Ring, and Finn Hallböök.

***Fig. S3C. Analysis of the fraction of MYCN-GFP in electroporated E14 retina***

Cells from the electroporated region of E14 retinas were dissected, dissociated and analyzed using fluorescence-activated cell sorting (FACS). The analysis shows the fraction of cells that received and express the piggyBack integration with MYCN-GFP. The fraction is the proportion of GFP positive cells compared to the total number of dissected retinal cells.

The gates are: “pop” for the cell population to remove debris; “siS” for cell population gated on side scatter; “siF” for single cell population gated on forward scatter; “GFP sort” for GFP positive single cells. In total 1890 GFP events out of 290 792 total events were gated giving a yield of 0.7% GFP positive cells


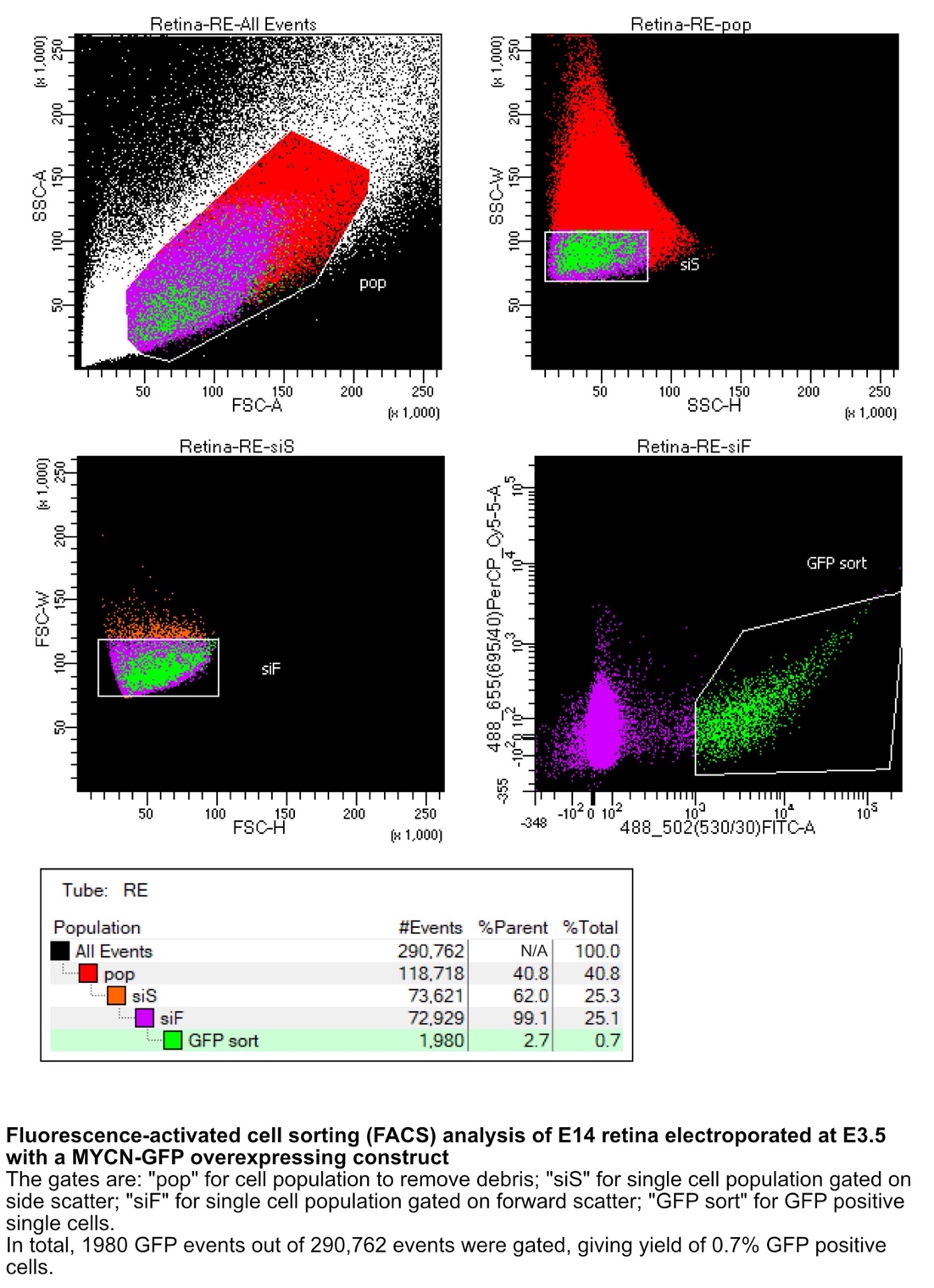

Supplement: Supplementary file 7 — Supplementary figure S3C [file 41389_2022_409_MOESM7_ESM.docx]
