# Supplementary material for: MYCN induces cell-specific tumorigenic growth in RB1-proficient human retinal organoid and chicken retina models of retinoblastoma
Source: Oncogenesis. 2022 Jun 21;11(1):34. doi: 10.1038/s41389-022-00409-3 (PMC9213451; doi:10.1038/s41389-022-00409-3)

Supplementary figure S3D

*MYCN* induces tumorigenic growth in *RB1*-proficient human retinal organoid- and chicken retina models of retinoblastoma.

Maria K E Blixt, Minas Hellsand, Dardan Konjusha, Hanzhao Zhang, Sonya Stenfelt, Mikael Åkesson, Nima Rafati, Tatsiana Tararuk, Gustav Stålhammar, Charlotta All-Eriksson, Henrik Ring, and Finn Hallböök.

***Fig. S3D. Tumours in chickens with MYCN/MYCN^T58A^-expression***

Eyes of chicken subjected to electroporation of *MYCN*-overexpressing vector at E3.5 as well as eyes that were injected with established *MYCN*^T58A^-expressing cells (orthotopic transplantation) at E5 or E16-18 were analysed. The animals were euthanized at the indicated ages and treated eyes were analysed using histology: haematoxylin to visualise the morphology and localization of tumour cells and immunohistochemistry with the antibodies indicated in the table below to characterise the neoplastic phenotype. Fourteen animals aged between P11 – 41 with transplanted *MYCN*^T58A^-cells and four *MYCN*-vector electroporated animals were analysed. All the tumours exhibited cell-type markers associated with cone/horizontal cell progenitor profile and none of the analysed animals exhibited markers associated with retinal ganglion or amacrine cells. Images exemplifies the localization of tumours in electroporated and cell transplanted eyes. There was heterogeneity in the tumour localization, particularly in eyes after orthotopic transplantation.

1. Fluorescence micrographs of eyes injected with MYCN T58A cells at E5 and analysed at E6 (top panel) or E14 (bottom panel) showing immunoreactivity for retinal cell type markers: Visinin (photoreceptors), Ap2α (amacrine cells) and Brn3a (ganglion cells).
2. Table summarizing localisation and expression of markers in MYCN-GFP+ cells in cell-injected and electroporated eyes. -; background levels, +; detectable, ++; clear, +++; robust fluorescent signal.
3. Bright-field micrographs of haematoxylin-stained sections from eyes electroporated with the MYCN-GFP overexpression vector constructs at E3.5 and analysed at 43- and 58 days post-hatch as indicated.
4. Micrographs from eyes that were orthotopically transplanted with MYCN^T58A^ cells. Numbers refer to the experimental animal sample id.

Abbreviations: eo; extraocular tumour, io; intraocular tumour, ir; iris, iret; intraretinal tumour, iv; intravitreal, le; lens, on; optic nerve, P; post-hatch day, re; retina, sc; sclera.

Scale bar in a) is 10µm. Scale bar in c) is also valid for d) and is 200µm.

a)


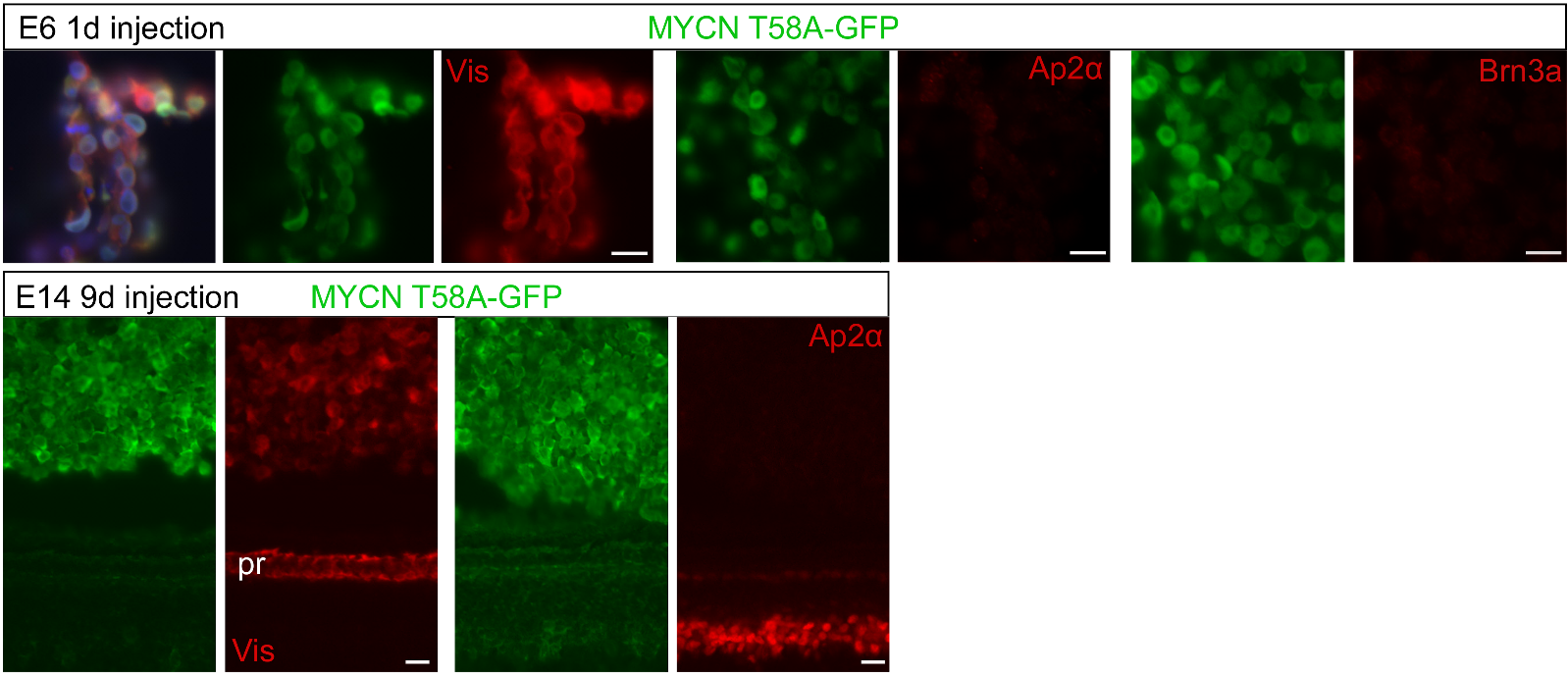


b)

***
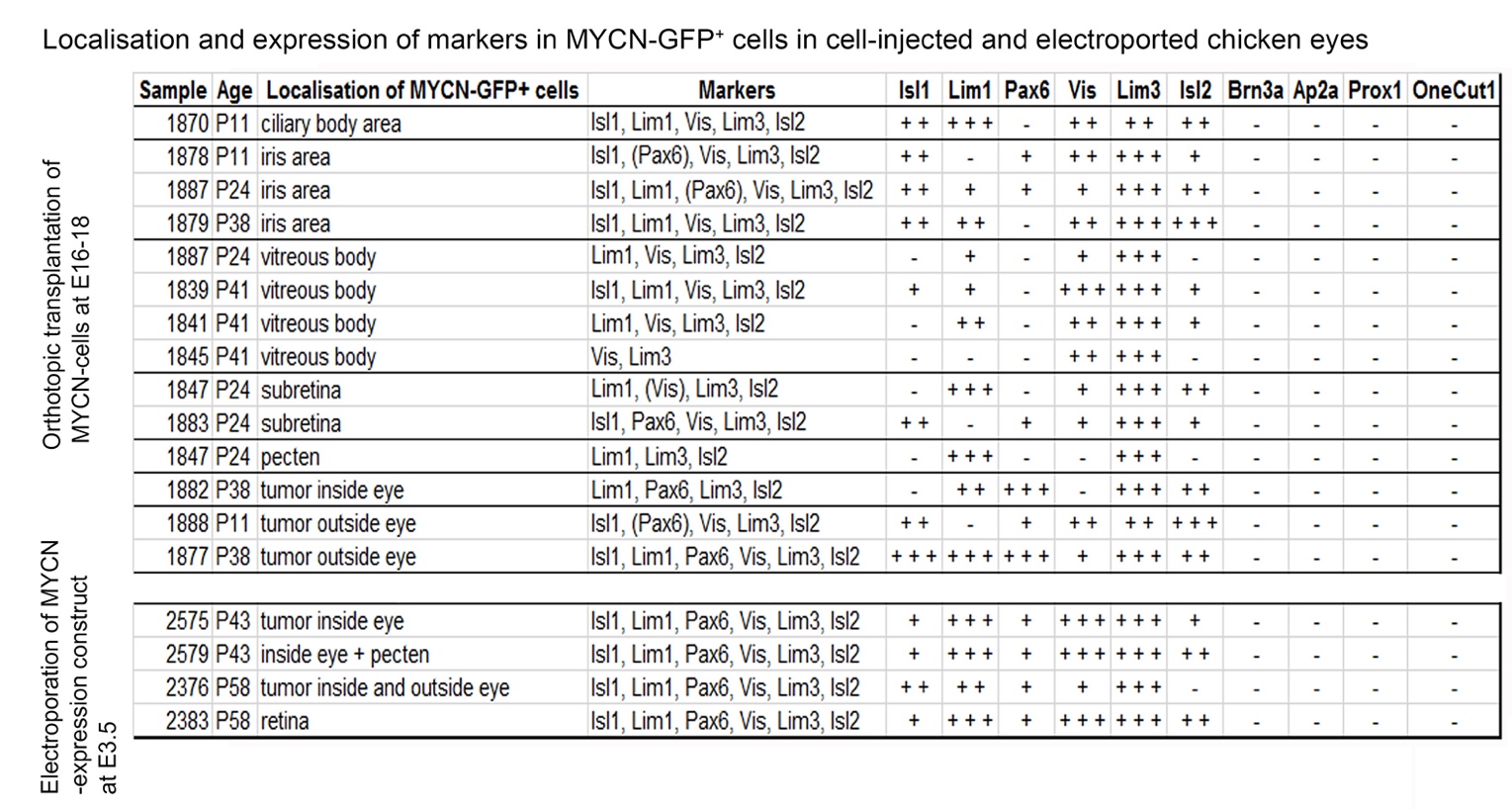
***

c)


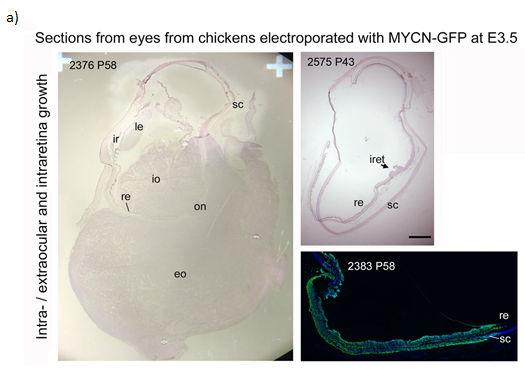


d)


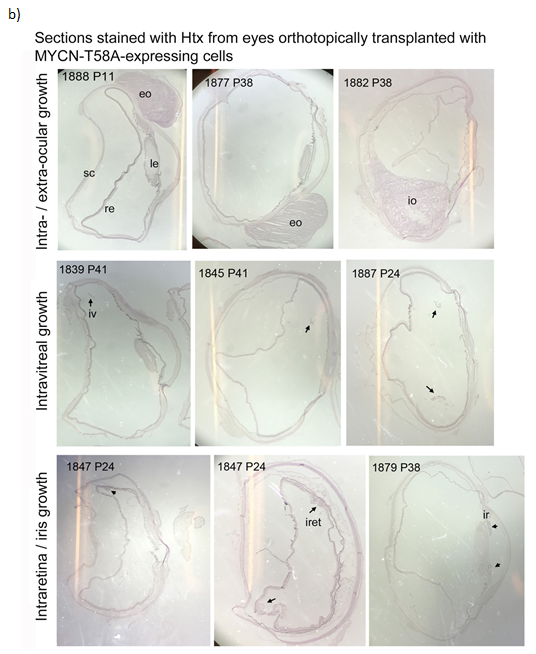

Supplement: Supplementary file 8 — Supplementary figure S3D [file 41389_2022_409_MOESM8_ESM.docx]
