# Supplementary material for: MYCN induces cell-specific tumorigenic growth in RB1-proficient human retinal organoid and chicken retina models of retinoblastoma
Source: Oncogenesis. 2022 Jun 21;11(1):34. doi: 10.1038/s41389-022-00409-3 (PMC9213451; doi:10.1038/s41389-022-00409-3)

Supplementary figure S4A

*MYCN* induces tumorigenic growth in *RB1*-proficient human retinal organoid- and chicken retina models of retinoblastoma.

Maria K E Blixt, Minas Hellsand, Dardan Konjusha, Hanzhao Zhang, Sonya Stenfelt, Mikael Åkesson, Nima Rafati, Tatsiana Tararuk, Gustav Stålhammar, Charlotta All-Eriksson, Henrik Ring, and Finn Hallböök.

***Fig. S4A. Complementary micrographs of naïve retinoids***

Fluorescence micrographs showing immunoreactivity for retinal cell type markers over time in naïve, non-electroporated retinoids. Several of the markers are associated with more than one cell subtype with independent developmental profiles: Onecut1 and Brn3 (note: pan-Brn3, which recognizes Brn3a, -b, and -c) for basally-located cell types, including retinal ganglion cells, Lim1 and Onecut2 for photoreceptor and horizontal cell progenitors, Ap2α for amacrine cells, Otx2 for cells in the photoreceptor lineage, Isl1 for subtypes of ganglion and amacrine cells, and Prox1 for horizontal cells. The expression pattern in the retinoids is consistent with development, though they are heterogeneous in size and structure. Abbreviations: ap; apical side, bas; basal side, inl; inner nuclear layer, os; outer photoreceptor segments, onl; outer nuclear layer. Scale bar is 25 μm and valid unless otherwise indicated.


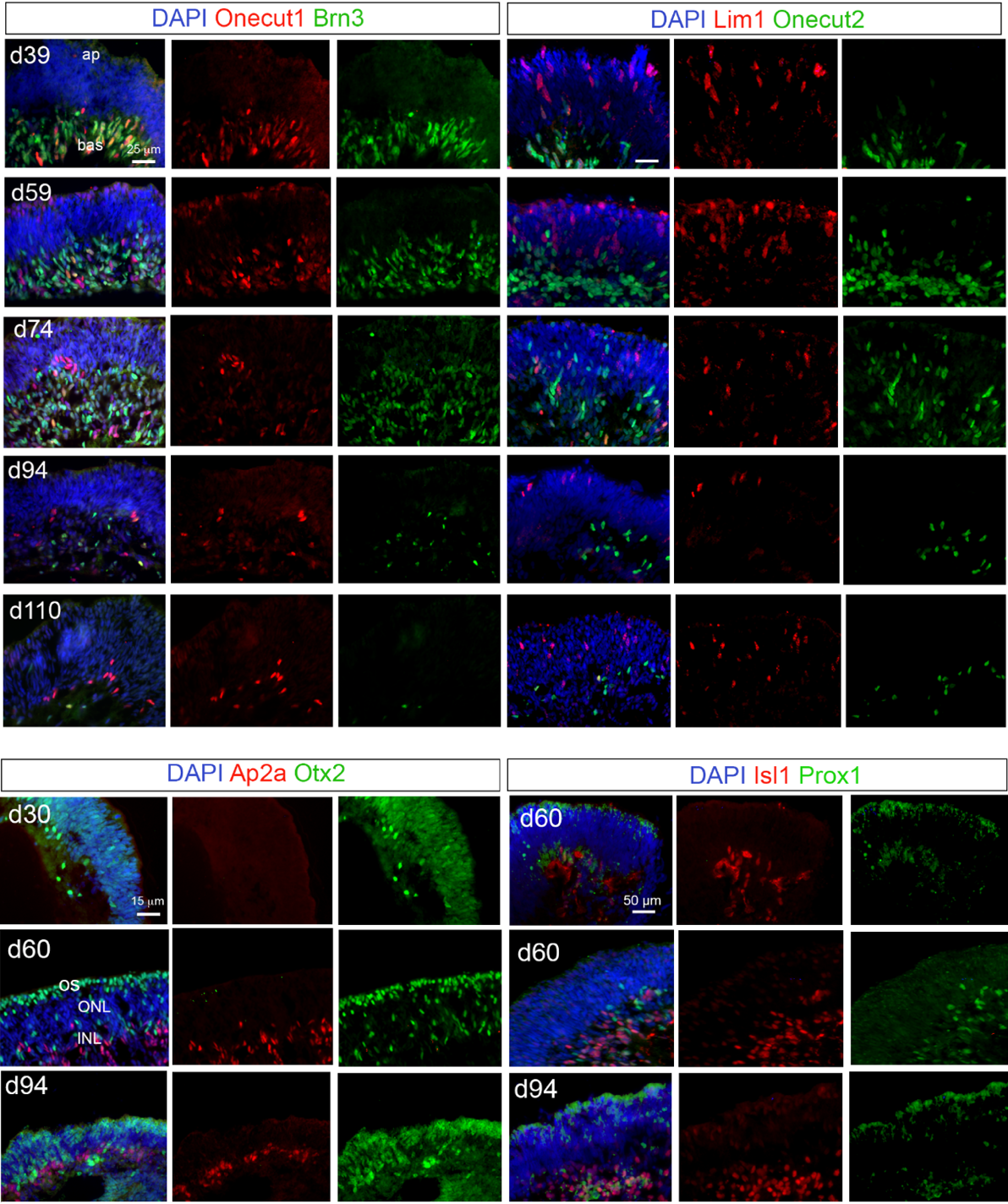

Supplement: Supplementary file 9 — Supplementary figure S4A [file 41389_2022_409_MOESM9_ESM.docx]
