# Supplementary material for: MYCN induces cell-specific tumorigenic growth in RB1-proficient human retinal organoid and chicken retina models of retinoblastoma
Source: Oncogenesis. 2022 Jun 21;11(1):34. doi: 10.1038/s41389-022-00409-3 (PMC9213451; doi:10.1038/s41389-022-00409-3)

Supplementary figure S4B

*MYCN* induces tumorigenic growth in *RB1*-proficient human retinal organoid- and chicken retina models of retinoblastoma.

Maria K E Blixt, Minas Hellsand, Dardan Konjusha, Hanzhao Zhang, Sonya Stenfelt, Mikael Åkesson, Nima Rafati, Tatsiana Tararuk, Gustav Stålhammar, Charlotta All-Eriksson, Henrik Ring, and Finn Hallböök.

***Fig. S4B. Scoring of “neoplastic stages” in MYCN expressing retinoids.***

Retinoids were electroporated at day 39-42 with piggyBac expression vectors that drive expression of MYCN-GFP, MYCN^T58A^-GFP or GFP under the control of a ubiquitous actin promotor. MYCN or MYCN^T58A^ and GFP are expressed from a bicistronic transcription unit and a GFP-only vector without MYCN or MYCN^T58A^ was used as control. The development of cell growth (neoplastic stages/phenotype) was monitored using GFP expression and was staged as described in Figure 4 and below. The stacked bar graphs and tables below them shows the number and ages of retinoids that were used for the scoring. The stage was scored from 0-5 where 0-1 is a normal appearance with scattered green cells and scores 2-5 have clusters of green cells that also disrupt the retinoid structure (stage 4-5). Such stages are indicated as “neoplastic“. Day; days in culture for retinoids, EB; embryoid body, hESCs; human embryonic stem cells, RC; retinal cup.


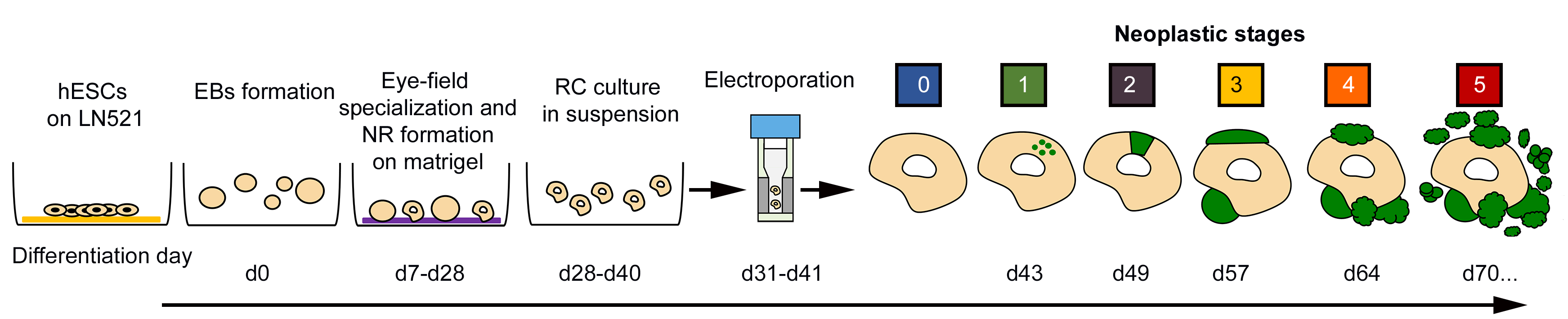


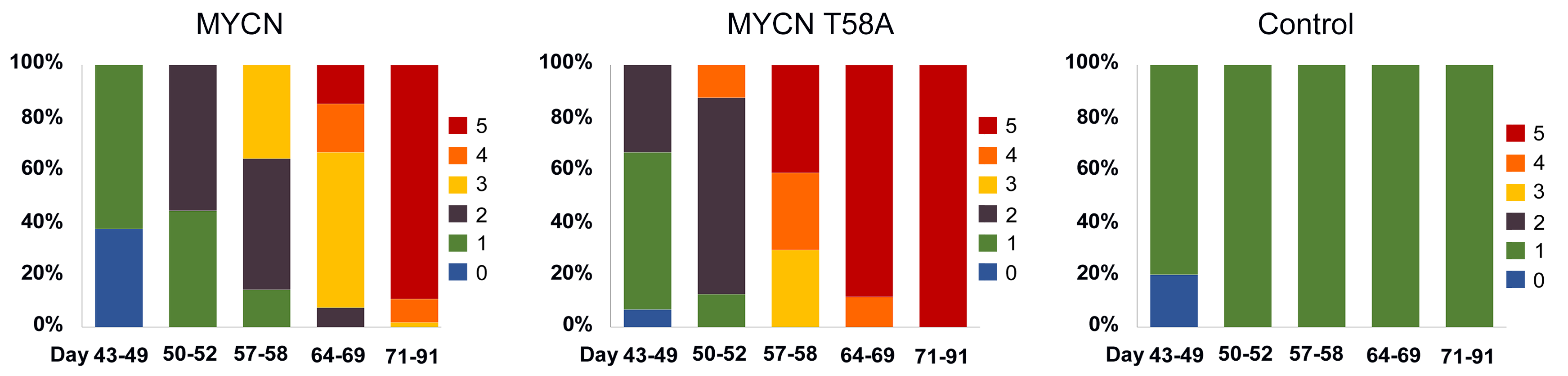


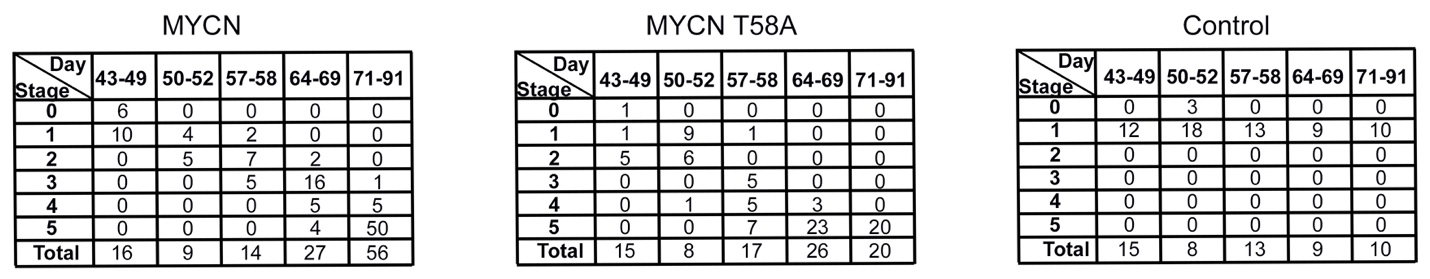

Supplement: Supplementary file 10 — Supplementary figure S4B [file 41389_2022_409_MOESM10_ESM.docx]
