# Supplementary material for: MYCN induces cell-specific tumorigenic growth in RB1-proficient human retinal organoid and chicken retina models of retinoblastoma
Source: Oncogenesis. 2022 Jun 21;11(1):34. doi: 10.1038/s41389-022-00409-3 (PMC9213451; doi:10.1038/s41389-022-00409-3)

Supplementary figure S5B

*MYCN* induces tumorigenic growth in *RB1*-proficient human retinal organoid- and chicken retina models of retinoblastoma.

Maria K E Blixt, Minas Hellsand, Dardan Konjusha, Hanzhao Zhang, Sonya Stenfelt, Mikael Åkesson, Nima Rafati, Tatsiana Tararuk, Gustav Stålhammar, Charlotta All-Eriksson, Henrik Ring, and Finn Hallböök.

***Fig. S5B. Complementary micrographs of MYCN^T58A^ retinoids stained for cell-type markers***

Fluorescence micrographs showing immunoreactivity for retinal cell type markers a) Otx2 (photoreceptors), b) Lim1 (Lim1/2; progenitors for photoreceptors and horizontal cells), c) Ap2α for amacrine cells, and d) pan-Brn3 (note: pan-Brn3 recognises Brn3a, -b, and -c) for retinal ganglion cells. Left panels in each row are in low magnification and dotted-line boxed regions are magnified in 3 panels to their right of each row. Arrowheads indicate examples of double-positive cells. Abbreviations: ap; apical side of retina, bas; basal side of retina, d; retinoid age (day). Scalebars (left top panels) in a)-d) are 100 µm and scale bars in magnified panels are 25 µm.


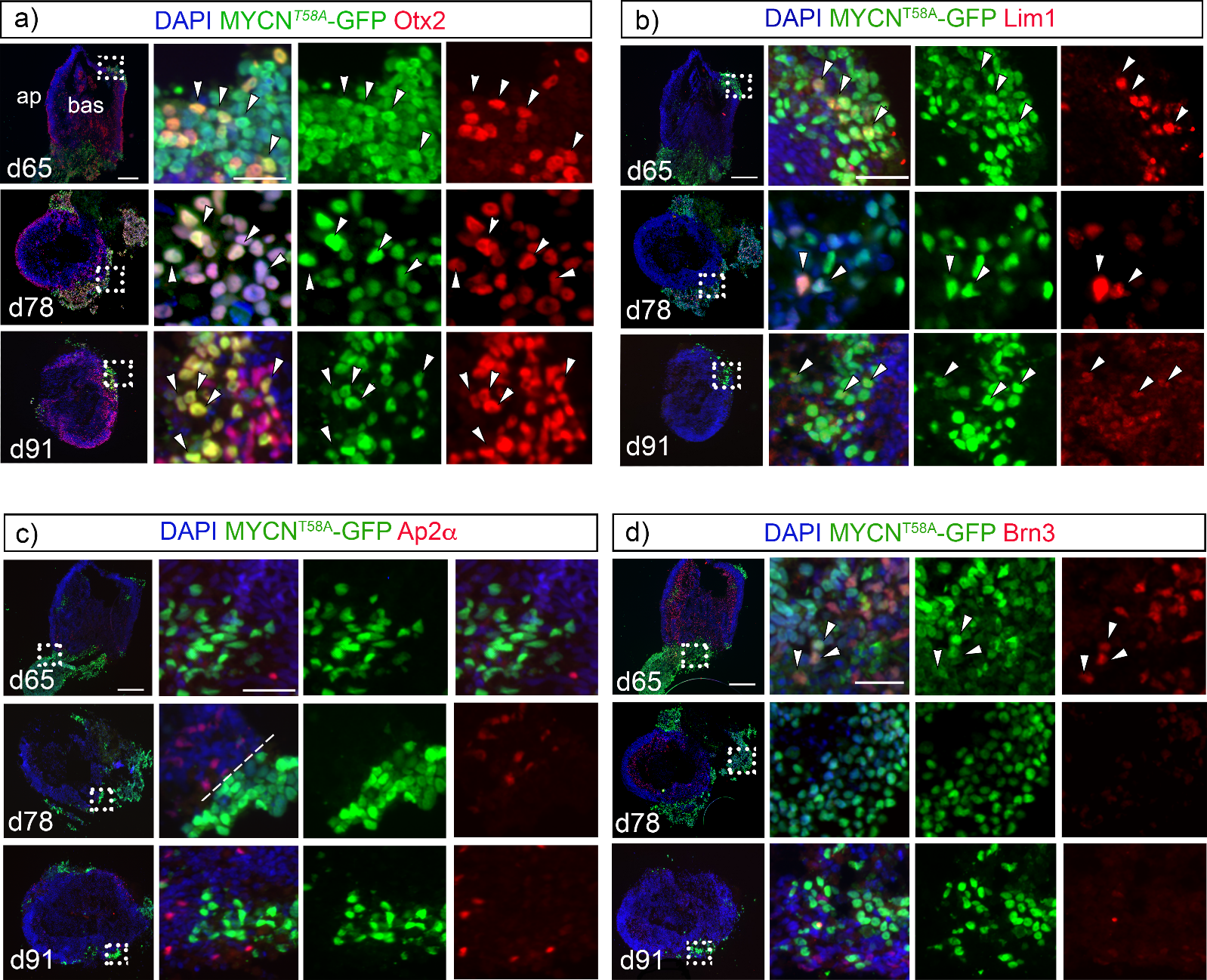

Supplement: Supplementary file 12 — Supplementary figure S5B [file 41389_2022_409_MOESM12_ESM.docx]
