# Supplementary material for: MYCN induces cell-specific tumorigenic growth in RB1-proficient human retinal organoid and chicken retina models of retinoblastoma
Source: Oncogenesis. 2022 Jun 21;11(1):34. doi: 10.1038/s41389-022-00409-3 (PMC9213451; doi:10.1038/s41389-022-00409-3)

Supplementary figure S6A

*MYCN* induces tumorigenic growth in *RB1*-proficient human retinal organoid- and chicken retina models of retinoblastoma.

Maria K E Blixt, Minas Hellsand, Dardan Konjusha, Hanzhao Zhang, Sonya Stenfelt, Mikael Åkesson, Nima Rafati, Tatsiana Tararuk, Gustav Stålhammar, Charlotta All-Eriksson, Henrik Ring, and Finn Hallböök.

***Fig. S6A. Complementary micrographs of control and MYCN-retinoids stained for RXRγ, Otx2 and Arrestin-3 (ARR3).***

Fluorescence micrographs showing immunoreactivity in retinoids of 74 to 111 days. a) Control, naïve retinoids and b) *MYCN*-GFP retinoids stained for RXRγ and Otx2. c) Control, naïve retinoids and d) *MYCN*-GFP retinoids stained for ARR3 and Otx2. Abbreviations: ap; apical part of retina, bas; basal part of retina, d; retinoid age (day). Scale bar in a) is 25 µm and applies to all micrographs.


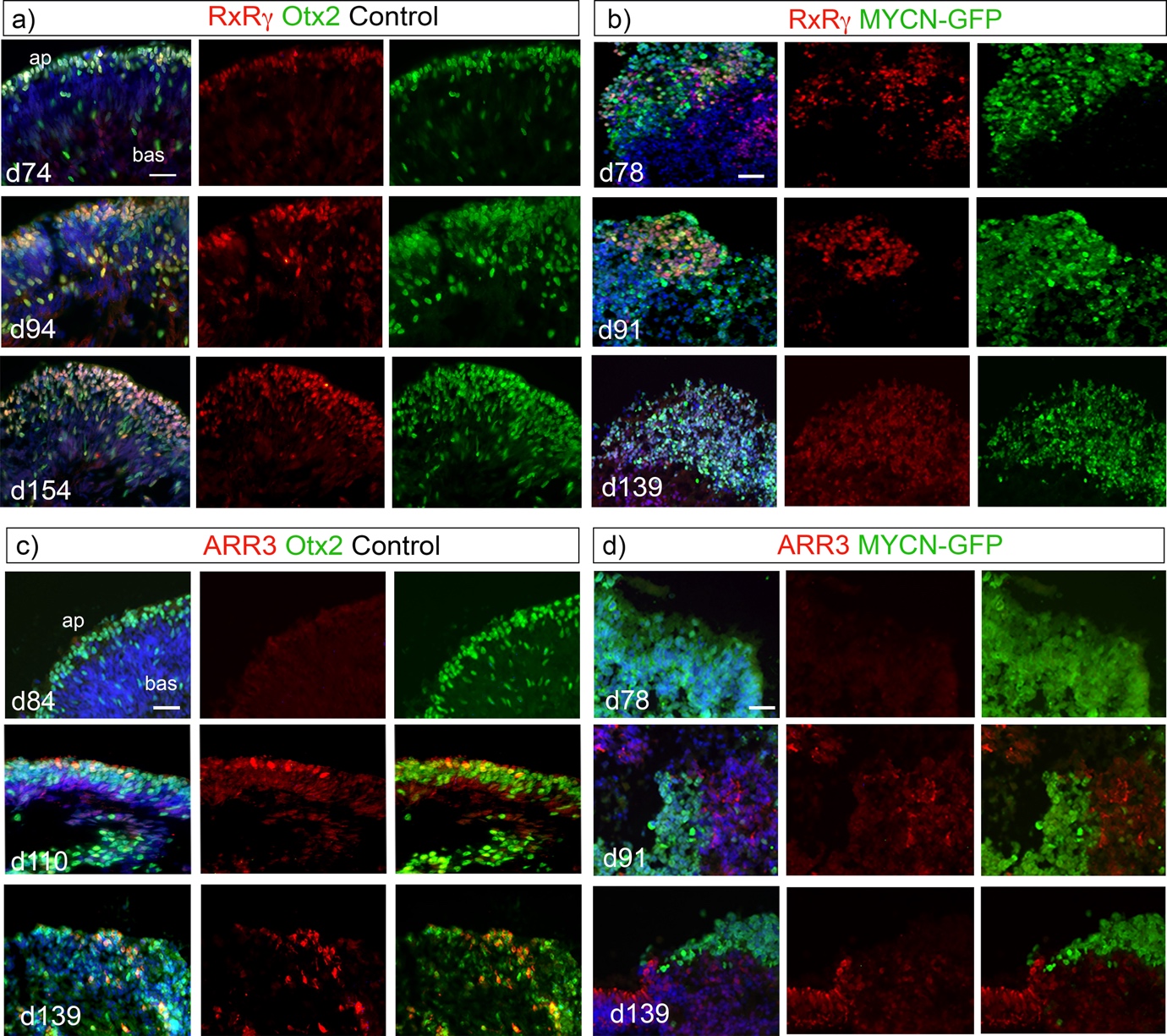

Supplement: Supplementary file 13 — Supplementary figure S6A [file 41389_2022_409_MOESM13_ESM.docx]
