# Supplementary material for: MYCN induces cell-specific tumorigenic growth in RB1-proficient human retinal organoid and chicken retina models of retinoblastoma
Source: Oncogenesis. 2022 Jun 21;11(1):34. doi: 10.1038/s41389-022-00409-3 (PMC9213451; doi:10.1038/s41389-022-00409-3)

Supplementary figure S6B

*MYCN* induces tumorigenic growth in *RB1*-proficient human retinal organoid- and chicken retina models of retinoblastoma.

Maria K E Blixt, Minas Hellsand, Dardan Konjusha, Hanzhao Zhang, Sonya Stenfelt, Mikael Åkesson, Nima Rafati, Tatsiana Tararuk, Gustav Stålhammar, Charlotta All-Eriksson, Henrik Ring, and Finn Hallböök.

***Fig. S6B. Complementary micrographs of MYCN-retinoids stained for Rb, Rb phospho-Serine 608(P-S608), Ki67 and PH3.***

Fluorescence micrographs show immunoreactivity in retinoids of 65 to 111 days. a) “Total” retinoblastoma protein, Rb, b) phospho-S608 Rb, RB(P-S608), c) proliferation antigen Ki67, and d) phospho-Histone 3, PH3. Arrowheads indicate examples of double-positive cells. Abbreviation d; retinoid age (day). Scalebar in a) is 25 µm and is also valid for b)-d).


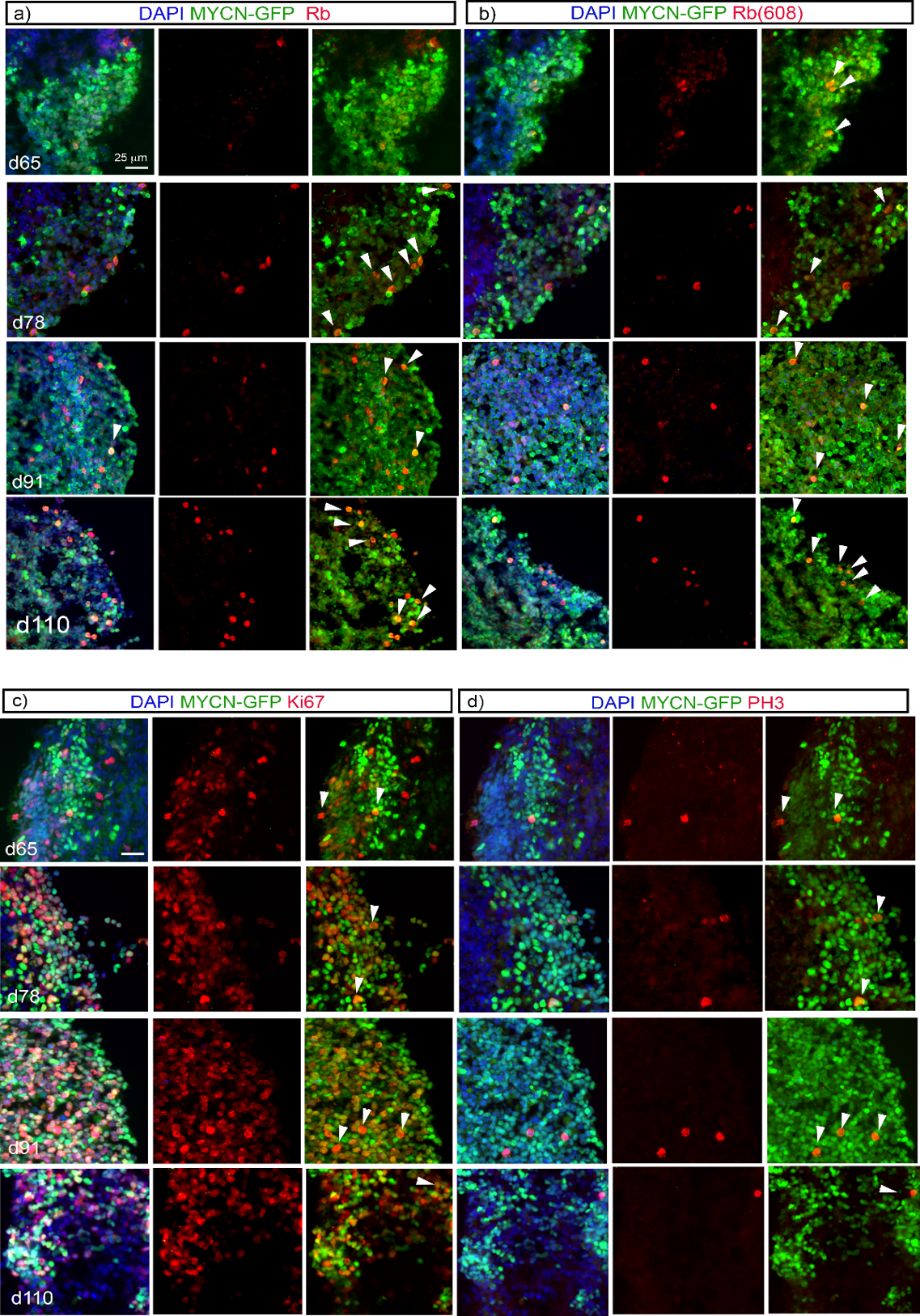

Supplement: Supplementary file 14 — Supplementary figure S6B [file 41389_2022_409_MOESM14_ESM.docx]
